# Joint Trust-Aware Topology Adaptation and Resource-Constrained Patching for Malware Containment in the Social Internet of Things

**DOI:** 10.3390/e28101078

**Published:** 2026-09-30

**Authors:** Xuejin Zhu, Shenyang Li

**Affiliations:** 1 School of Internet of Things Engineering, Wuxi University, Wuxi 214105, China; 2 School of Computer Science, Nanjing University of Information Science & Technology, Nanjing 210044, China

**Keywords:** malware propagation, Social Internet of Things, adaptive networks, optimal control, spectral threshold, trust management

## Abstract

Social Internet of Things (SIoT) relationships enable service discovery and create pathways for malware propagation. We develop a heterogeneous susceptible–exposed–infected–recovered model coupling resource-constrained patching with reversible, trust-aware relationship suppression. A controlled next-generation operator yields a reproduction threshold, local stability, spectral monotonicity, and a uniform eradication condition. A finite-horizon security–service objective and its necessary conditions motivate joint adaptive topology and patching (JATP), a feasible sampled-data feedback heuristic combining prevalence, structural influence, trust evidence, and service importance. Under reference capacities, JATP reduces peak infection and cumulative burden by 49.43% and 38.97% relative to risk-only patching while retaining 0.8184 of weighted relationship intensity. Under a common expenditure ceiling, its objective is 59.56% above the best feasible offline nonlinear-programming value found. Explicit-network tests support aggregation under moderate heterogeneity and identify larger prediction errors under strong degree variation and susceptible-entry churn. Trust-related gains depend on alignment with model-defined propagation risk; field calibration remains open. These results characterize the operating scope of a feedback policy that coordinates two defense channels under hard resource limits. The framework links epidemic thresholds, intervention allocation, and service preservation to support the design of resource-limited SIoT containment.

## 1. Introduction

The Internet of Things (IoT) has evolved from collections of isolated sensors into heterogeneous ecosystems of service-providing devices. In the Social Internet of Things (SIoT), objects form relationships analogous to human social ties and use local friend-of-friend navigation for service discovery [1,2]. Ownership, co-location, co-work, parental-object, and owner-social relationships make a large device population navigable without a centralized directory. This architectural advantage has a security cost. Malware does not propagate over a neutral substrate: it follows semantically meaningful, repeatedly used, and sometimes highly trusted paths. A compromised gateway or hub can therefore connect communities that would otherwise interact weakly.

Self-propagating malware remains difficult to analyze because scanning, latent execution, dormant behavior, and remediation occur on different time scales. Classical directed-graph models established the connection between computer viruses and epidemic dynamics [3,4]. Network epidemiology subsequently demonstrated that degree heterogeneity and spectral structure determine outbreak thresholds [5,6]. Recent empirical work fitted compartmental dynamics to controlled WannaCry traces and showed that a model containing infectious and dormant states can outperform standard SI, SIS, and SIR descriptions [7]. Individual-based NIMFA formulations now permit latency and device-level topology to be studied simultaneously [8]. These advances make the epidemic abstraction increasingly useful, but most analyses still treat the contact graph as fixed.

Fixed connectivity is particularly restrictive in SIoT. A social relationship is a logical policy object, not an immutable physical edge. A platform can reduce its weight, rate-limit it, require renewed authentication, replace its service provider, or remove it temporarily. Trust can also change with context and behavior [9,10,11,12], which motivates a policy that can accept updated external scores. In the present numerical study, trust evidence is exogenous and time-invariant within each run; a controlled quality experiment evaluates how its alignment with latent relationship risk affects allocation.

Patching provides a second control channel. Optimal patching can exploit clusters and device heterogeneity [13]. Traffic-aware schemes protect intermediate nodes that lie on many infection paths [14]. More recent studies use optimal control, model predictive control, game theory, distributed patch dissemination, or reinforcement learning to balance infection and intervention costs [15,16,17,18,19]. However, patch production, gateway bandwidth, energy, maintenance windows, and reboot tolerance impose hard capacity limits. Directing every patch to the weakest devices is not necessarily optimal because a better-protected hub can cause more secondary infections if compromised. Conversely, isolating every high-degree object destroys reachability and undermines the purpose of SIoT.

Against this background, the literature needed to formulate a joint security–service response spans three connected research streams. Early malware models abstracted hosts as susceptible and infected nodes on directed graphs [3,4]. Later work added exposed, quarantined, vaccinated, dormant, recovered, and temporarily immune states. A broad survey by Wang et al. [6] organizes these models by propagation mechanism, network structure, and analytical method. For wireless sensor networks (WSNs), sleep schedules, communication range, energy depletion, and cluster heads influence propagation [20,21]. A heterogeneous IoT formulation distinguished latent and propagating devices across device types and optimized recovery allocation [22]. A hierarchical-protection model further showed that highly protected nodes may remain consequential because their connectivity amplifies the impact of rare compromise [23].

Recent research improves realism along four directions. First, data-grounded modeling uses observed attack traces rather than only synthetic curves [7]. Second, individual-based mean-field approximations preserve node-level topology while avoiding the full Markov-chain state explosion [8]. Third, propagation prediction combines learned node representations with graph convolution [24]. Fourth, non-classical dynamics represent memory, multiple infections, time delay, or noise. Examples include fractional-order malware models with optimal control [25], explicit external noise and multiple malware infections [26], and delayed distributed patch propagation [18]. These contributions motivate our uncertainty and delayed-observation experiments. Our focus differs: we retain an interpretable compartmental state but make the relationship operator itself a feedback-controlled variable.

A second stream studies node recovery, vaccination, quarantine, awareness, patch dissemination, and spatial feedback. Clustered optimal patching formalizes resource allocation over heterogeneous groups [13]; traffic-aware patching protects nodes that relay many malicious flows [14]. Recent optimal-control studies distinguish stages of infection and immediate pre-patch response [15], or minimize antivirus installation cost on a virus-threatened network [27]. Security awareness changes both infection and treatment behavior in mobile networks [28], while physics-informed neural networks can estimate epidemic parameters before selecting IoT nodes for mitigation [29].

Control is also moving from ideal open-loop trajectories toward implementable feedback. An RL–MPC roadmap learns and updates malware controls online [16]; attack–defense games capture strategic allocation in cloud environments [17]; and reinforcement learning has been used for channel-aware mitigation in radar sensor networks [19]. A recent hierarchical multi-agent framework for moving-target defense in cyber–physical systems further demonstrates the value of separating strategic adaptation, distributed allocation, and safe execution under resource constraints [30]. Hybrid knowledge- and data-driven control has been studied for coupled underground and aboveground sensor networks [31], and for cyber–physical malware processes [32]. Spatiotemporal feedback controllers address outbreak hotspots and time delay [33]. Mobile IoT work combines a temporal multilayer network, device diffusion, competing patch propagation, and capacity-constrained patch allocation [34]. These studies strongly support adaptive control, yet relation-level service loss usually remains outside the objective. JATP explicitly coordinates recovery with logical-topology intervention and measures the retained service substrate.

Topology-aware containment also appears in spatial-firewall and behavioral models. Percolation analysis links the density and placement of defenses to the existence of a giant vulnerable component [35]. Multiplex-network research integrates layer importance, intra-layer structure, and projected centrality to identify key spreaders [36], but node ranking alone does not determine a feasible time-varying defense. In SIoT, the STSIR model combines individual and group games to describe how strategic behavior changes virus spreading [37]. These studies reinforce the importance of endogenous contact decisions and motivate evaluating reversible relationship suppression and patching jointly with an explicit service criterion.

A third stream concerns SIoT trust and relationship management. The foundational SIoT architecture treats devices as social objects capable of autonomously forming relationships [1]. Friendship-selection mechanisms influence hub concentration, clustering, average path length, and navigability [2]. Trust management then ranks providers and reduces interaction with unreliable objects [38]. Contemporary reviews show that trust computation spans direct experience, recommendations, context, social similarity, uncertainty, and attack resistance [9,10]. Trust also has safety, fairness, transparency, and accountability dimensions when AI participates in autonomous decisions [39].

Several recent systems address adversarial or dynamic evidence. A resilient SIoT framework explicitly considers attacks against the trust-evaluation process [40]. Fuzzy multi-criteria modeling ranks trust metrics and submetrics [41]. Role-based intra- and inter-community evaluation separates service-provider, rater, and community cooperation evidence [42]. Self-adaptive trust integrates MAPE-K control, fog computing, federated learning, and changing hostility thresholds [11]. REACT-SIoT provides a more realistic evaluation platform for dynamic trust conditions [43], while cluster-level trust dynamicity captures continuous behavioral and contextual change [12]. Zero-trust research reinforces the principle that a prior relationship should not imply unlimited future access [44].

JATP receives relationship-level evidence from an external trust engine at each control epoch. This evidence, together with estimated malware prevalence, determines a reversible transmission multiplier. Separating trust estimation from epidemic control allows JATP to consume direct, recommendation-based, zero-trust, or learned scores without changing the epidemic state equations.

Taken together, these streams motivate a joint treatment of epidemic control and service-dependent relationship adaptation. Table 1 compares representative recent formulations in terms of topology and modeling granularity, defense channels, trust information, service criteria, feedback and solution methods, and analytical thresholds. The present model combines a controlled reproduction threshold, shared patch and relationship capacities, and an explicit retained-intensity penalty. The contribution is this joint formulation and the evaluation of a feasible feedback policy within it.

This paper therefore asks: how should a resource-limited SIoT jointly allocate patches and adapt relationships so that malware is contained without disabling service discovery? We answer through a heterogeneous adaptive-network epidemic model and an implementable feedback policy. The relation layer is controlled through reversible suppression rather than permanent deletion. A retained-relationship penalty and an explicit shared suppression budget make aggregate service loss costly and prevent unconstrained shutdown in the implemented controller. The patch and topology actions are coupled through current infection pressure, independent trust evidence, and structural influence.

Six research questions guide evaluation. RQ1 compares JATP with patch-only, topology-only, and numerical optimization under channel-specific capacities and a common normalized intervention cost. RQ2 measures relationship-capacity and graph-level service loss. RQ3 tests whether individually insufficient, budget-feasible actions jointly cross the initial-state spectral threshold. RQ4 examines resource, feedback, trust, contact, and topology sensitivity. RQ5 assesses parameter uncertainty, controller period, delayed/noisy observations, and malicious telemetry. RQ6 tests explicit static and time-varying networks and computational and communication scaling.

The principal contributions are as follows.

1. A *K*-stratum SEIR model couples heterogeneous protection, latency, immunity loss, susceptible patching, infected repair, and trust-aware relationship suppression under shared resource constraints.
2. Positivity, invariance, a controlled next-generation matrix, local threshold stability, spectral monotonicity, and a sufficient uniform-eradication condition under time-varying controls characterize the dynamics. A finite-horizon security–service optimal-control problem yields necessary conditions coupling patching and relationship actions.
3. JATP converts prevalence estimates, external edge trust, and topology-specific Perron influence into feasible sampled-data actions. It enforces population-weighted patch capacity and a shared suppression budget, accounts for service-dependent costs, and has $O(K^{2})$ online complexity.
4. Numerical comparisons with patch-only feedback and a feasible offline optimization benchmark quantify containment and service costs. Trust quality, telemetry, network heterogeneity, and susceptible-entry churn identify conditions affecting control performance and aggregate prediction accuracy.

The remainder of the paper presents the threat model, dynamics, threshold theory, optimal-control formulation, and feedback design in Section 2; specifies the simulation protocol and reports the numerical results in Section 3; discusses mechanisms, deployment implications, and validity threats in Section 4; and concludes in Section 5. Detailed derivations and additional robustness results are provided in the appendices.

## 2. Materials and Methods

This section specifies the managed SIoT threat model, the adaptive heterogeneous propagation process, its spectral threshold, the joint control problem, and the deployable feedback policy.

### 2.1. Threat Model and Design Objectives

We consider a managed SIoT domain such as a smart campus, industrial facility, healthcare complex, or large smart-home federation. Devices expose services and maintain logical social relationships. A relationship permits discovery or communication but can be rate-limited or suspended by a gateway, service mesh, or policy controller. Devices are grouped into *K* strata according to role, software stack, protection level, or administrative policy. Aggregation is appropriate when per-device state is unavailable or when a controller must operate at deployment scale.

The endpoint–gateway–hub instance represents three functional roles. Endpoints are numerous service consumers, gateways aggregate local domains, and hubs provide highly reused discovery or coordination services. The class matrix retains asymmetric cross-class exposure and critical-node influence through its entries and Perron weights; within-class degree, community, and vulnerability variation is aggregated. Section 3 compares the system with explicit 100–1000-node graphs containing degree and vulnerability variation, periodic relationship regeneration, and node replacement. Deployments with finer role or community information can increase *K* without changing the equations.

The attacker obtains an initial foothold through an unpatched vulnerability, weak credential, malicious update, or compromised neighbor. Infectious devices attempt to propagate along active relationship classes. An exposed device contains malware but has not yet begun outward propagation; this represents activation latency, command-and-control delay, or dormant execution. The attacker does not directly control the defender’s patch server, but may exploit highly trusted or central objects and may indirectly distort trust evidence; strategic time-varying attacks on trust estimation remain outside this study. We test observation delay, multiplicative state-estimation error, and a controlled trust-quality continuum ranging from reversed to perfectly aligned evidence in a latent-edge-risk scenario.

The defender has two actions. Patching intensity $u_{i}\left(t\right)$ immunizes susceptible devices and repairs infectious devices in stratum *i*. Relationship suppression $v_{ij}\left(t\right)$ reduces effective contacts from stratum *j* to *i*. Suppression may be implemented by rate limiting, renewed authentication, temporary quarantine, replacement of a service provider, or removal of a friendship edge. It is reversible, box constrained, and subject to a shared weighted budget. The control changes contact intensity continuously within each relationship class.

The defender observes stratum-level exposed and infected estimates at discrete control epochs. Estimates can combine endpoint alerts and propagation-flow anomalies, while an external trust engine supplies distinct edge-level evidence. Payload visibility is not required. Perron influence is computed offline from the actual baseline relationship matrix used in each scenario. Patches and relation actions have separate capacity limits; a retained-relationship penalty couples them in the objective.

Administrators should derive service importance before incidents from service catalogs, dependency graphs, request volume, recovery-time objectives, and criticality tiers, then normalize positive weights by their mean. Trust requires separate calibration: isotonic or logistic mappings can convert reputation or anomaly scores to reliable-behavior probabilities using temporally held-out incident logs, with calibration error and subgroup drift monitored. The reference matrices in this study are synthetic.

An operational policy should satisfy five requirements. It must: (i) reduce peak load because simultaneous compromise stresses incident response; (ii) reduce cumulative infected time because dwell time drives loss; (iii) respect patch bandwidth and reboot capacity; (iv) preserve weighted relationship capacity used for service discovery; and (v) remain useful with imperfect state estimates. These requirements motivate the running and terminal infection terms in the mathematical objective and the broader evaluation protocol; peak infection is evaluated separately and is not an explicit term in *J*.

### 2.2. Adaptive Heterogeneous Propagation Model

Let $N_{i}>0$ be the normalized population of stratum *i*, with $\sum_{i=1}^{K}N_{i}=1$. At time *t*, $S_{i},E_{i},I_{i},$ and $R_{i}$ denote susceptible, exposed, infectious, and recovered fractions. Each stratum satisfies

(1) $$S_{i}\left(t\right)+E_{i}\left(t\right)+I_{i}\left(t\right)+R_{i}\left(t\right)=N_{i}.$$

The baseline relationship matrix is $A^{0}=\left[a_{ij}^{0}\right]\geq 0$. An entry aggregates active-relation density, contact frequency, and protocol opportunity from infectious stratum *j* to susceptible stratum *i*. Defense suppresses a fraction $v_{ij}\left(t\right)\in \left[0,{\bar{v}}_{ij}\right]$, giving

(2) $$a_{ij}\left(t\right)=a_{ij}^{0}\left[1-v_{ij}\left(t\right)\right].$$

Relationships may be asymmetric; the reference matrix is symmetric because strata exchange services reciprocally. We use malware-free equilibrium (MFE) for the infection-free equilibrium. Table 2 lists the principal symbols.

Let $\beta_{i}$ be the receiving stratum’s effective transmission coefficient, $\sigma_{i}$ the exposed-to-infectious rate, $\gamma_{i}$ the native recovery rate, and $\omega_{i}$ the immunity-loss rate. The force of infection is

(3) $$\lambda_{i}\left(t\right)=\beta_{i}\sum_{j=1}^{K}a_{ij}^{0}\left[1-v_{ij}\left(t\right)\right]\frac{I_{j}\left(t\right)}{N_{j}}.$$

Patching intensity $u_{i}\left(t\right)\in \left[0,{\bar{u}}_{i}\right]$ protects susceptible devices and repairs infectious devices. The dynamics are

(4) $$\begin{array}{cc} {\dot{S}}_{i} & =-\lambda_{i}S_{i}-u_{i}S_{i}+\omega_{i}R_{i}, \end{array}$$

(5) $$\begin{array}{cc} {\dot{E}}_{i} & =\lambda_{i}S_{i}-\sigma_{i}E_{i}, \end{array}$$

(6) $$\begin{array}{cc} {\dot{I}}_{i} & =\sigma_{i}E_{i}-\left(\gamma_{i}+u_{i}\right)I_{i}, \end{array}$$

(7) $$\begin{array}{cc} {\dot{R}}_{i} & =\left(\gamma_{i}+u_{i}\right)I_{i}+u_{i}S_{i}-\omega_{i}R_{i}. \end{array}$$

The $u_{i}S_{i}$ term represents proactive security updating, immunization, or hardening that removes a susceptible device from immediate risk; $u_{i}I_{i}$ represents verified remediation, reimaging, or repair after compromise. Native recovery $\gamma_{i}I_{i}$ denotes remediation that occurs without the centrally allocated patch action, including autonomous rollback or routine incident response. If a deployment cannot patch an infected device without reimaging, different coefficients can multiply the two controlled terms without changing the analytical approach. No removal is placed in the exposed compartment in the reference model because dormant malware is assumed undetectable until activation. This makes every exposure progress to infectiousness and is the reason $\sigma_{i}$ cancels from the threshold; an exposed-stage detection rate is treated explicitly after Equation (10).

Equations (4)–(7) therefore have a direct flow interpretation. New compromise transfers mass from $S_{i}$ to $E_{i}$; activation transfers it from $E_{i}$ to $I_{i}$; native or controlled remediation transfers it from $I_{i}$ to $R_{i}$; proactive patching transfers it from $S_{i}$ to $R_{i}$; and immunity loss returns it from $R_{i}$ to $S_{i}$. These paired transfers also explain the conservation property below.

The relationship control and patch control act at different points in the infection chain. Increasing $v_{ij}$ prevents new exposures caused by $I_{j}$, whereas increasing $u_{i}$ removes infectious sources and reduces the susceptible pool. A joint strategy can therefore interrupt both the creation and persistence of infectious devices.

**Proposition** **1** (Existence, nonnegativity, and conservation)**.** *For measurable bounded controls and nonnegative initial conditions satisfying Equation (1), system (4)–(7) has a unique nonnegative solution. The compact region*

$$\Omega =\{\left(S,E,I,R\right)\geq 0:S_{i}+E_{i}+I_{i}+R_{i}=N_{i},\;i=1,\ldots ,K\}$$

*is positively invariant.*

**Proof.**  For bounded controls the vector field is measurable in time, locally Lipschitz in the state, and bounded on compact sets, so a unique Carathéodory solution exists. At $S_{i}=0$, ${\dot{S}}_{i}=\omega_{i}R_{i}\geq 0$; at $E_{i}=0$, ${\dot{E}}_{i}=\lambda_{i}S_{i}\geq 0$; at $I_{i}=0$, ${\dot{I}}_{i}=\sigma_{i}E_{i}\geq 0$; and at $R_{i}=0$, ${\dot{R}}_{i}=\left(\gamma_{i}+u_{i}\right)I_{i}+u_{i}S_{i}\geq 0$. Thus no coordinate crosses the nonnegative boundary. Summing Equations (4)–(7) gives zero for each stratum. Conservation and nonnegativity imply boundedness and positive invariance.   □

### 2.3. Threshold and Stability Analysis

For constant controls *u* and *v*, the malware-free equilibrium is

(8) $$E_{0}={\left(S_{i}^{*},0,0,R_{i}^{*}\right)}_{i=1}^{K},\;S_{i}^{*}=\frac{N_{i}\omega_{i}}{\omega_{i}+u_{i}},\;R_{i}^{*}=N_{i}-S_{i}^{*}.$$

Define the nonnegative new-exposure matrix *F* and infectious-removal matrix *D* by

(9) $$F_{ij}=\frac{\beta_{i}S_{i}^{*}a_{ij}^{0}\left(1-v_{ij}\right)}{N_{j}},\;D=\mathrm{diag}\left(\gamma_{i}+u_{i}\right).$$

The infected subsystem has exposed and infectious blocks. Every exposed device eventually enters the infectious state because $\sigma_{i}>0$ and there is no competing exposed removal. Consequently, the progression probability is one and the next-generation matrix reduces to

(10) $$K\left(u,v\right)=FD^{-1},\;R_{0}\left(u,v\right)=\rho \left(FD^{-1}\right).$$

Although $\sigma_{i}$ affects growth speed and peak timing, it cancels from this threshold under certain progression. If exposed devices can be detected and removed, the threshold gains the factor $\sigma_{i}/\left(\sigma_{i}+q_{i}\right)$.

The controlled MFE threshold in Equation (10) must be distinguished from a frozen-state index used to diagnose transient outbreak conditions. For an arbitrary susceptible vector *S*, define

(11) $$R_{\mathrm{inst}}\left(S;u,v\right)=\rho \;\left[\mathrm{diag}\left(\beta_{i}S_{i}\right)\left(A^{0}⊙\left(1-v\right)\right)\mathrm{diag}\left(N_{i}^{-1}\right)D{\left(u\right)}^{-1}\right].$$

This index counts secondary infections with susceptible fractions and controls frozen over the corresponding infectious lifetimes. It equals $R_{0}$ only at $S=S^{*}\left(u\right)$. The numerical grid uses $R_{\mathrm{inst}}\left(N;u,v\right)$, describing an initially fully susceptible state before proactive patching depletes susceptibility.

**Theorem** **1** (Local threshold)**.** *Assume the effective relationship graph is irreducible. The malware-free equilibrium is locally asymptotically stable if $R_{0}\left(u,v\right)<1$ and unstable if $R_{0}\left(u,v\right)>1$.*

**Proof.**  Linearizing (E,I) at $E_{0}$ gives

$$\left[\begin{array}{c} \dot{E}\\ \dot{I} \end{array}\right]=\left[\begin{array}{cc} -\Sigma & F\\ \Sigma & -D \end{array}\right]\left[\begin{array}{c} E\\ I \end{array}\right],\;\Sigma =\mathrm{diag}\left(\sigma_{i}\right).$$

This is a Metzler matrix. Applying the next-generation decomposition to new infections in *E* and transitions through *E* and *I* yields $FD^{-1}$. Standard positive-system threshold theory gives negative spectral abscissa precisely when $\rho (FD^{-1})<1$.   □

**Proposition** **2** (Control monotonicity)**.** *If ${\tilde{u}}_{i}\geq u_{i}$ and ${\tilde{v}}_{ij}\geq v_{ij}$ for all i,j, then $R_{0}\left(\tilde{u},\tilde{v}\right)\leq R_{0}\left(u,v\right)$. If the controlled next-generation matrix is irreducible and at least one positive entry is strictly reduced, the inequality is strict.*

**Proof.**  Increasing *v* decreases *F* entrywise. Increasing $u_{i}$ decreases both $S_{i}^{*}$ and the corresponding infectious duration ${\left(\gamma_{i}+u_{i}\right)}^{-1}$. Thus the controlled next-generation matrix decreases entrywise. The spectral radius of a nonnegative matrix is monotone under the componentwise order; irreducibility gives strictness for an effective change.   □

Let x,y>0 be right and left Perron eigenvectors of K, normalized by $y^{T}x=1$. For a simple dominant eigenvalue,

(12) $$\frac{\partial R_{0}}{\partial v_{ij}}=-y_{i}\frac{\beta_{i}S_{i}^{*}a_{ij}^{0}}{N_{j}\left(\gamma_{j}+u_{j}\right)}x_{j}.$$

Equation (12) makes a key point: the most valuable relation to suppress is not necessarily the largest $a_{ij}^{0}$. Its marginal value also depends on receiving susceptibility, infectious duration, and the left/right Perron weights that encode downstream and upstream amplification. The derivative with respect to $u_{i}$ contains both susceptible-depletion and infectious-duration effects. This explains why protection level, current prevalence, and centrality should be combined rather than used as isolated heuristics.

For feedback controls, define the linear comparison matrix

(13) $$M\left(t\right)=\left[\begin{array}{cc} -\Sigma & \bar{F}\left(t\right)\\ \Sigma & -D(t) \end{array}\right],$$

where $\bar{F}\left(t\right)$ is obtained from Equation (9) using an upper bound on susceptible fractions. An instantaneous spectral radius below one is informative but, by itself, need not guarantee stability under arbitrary switching. We therefore state a directly verifiable common-vector condition.

**Theorem** **2** (Uniform eradication under feedback)**.** *Suppose there exist $T_{0}\geq 0$, a vector p≫0, and δ>0 such that*

(14) $$M{\left(t\right)}^{T}p\leq -\delta p$$

*componentwise for almost every $t\geq T_{0}$. Then $E_{i}\left(t\right)$ and $I_{i}\left(t\right)$ converge to zero exponentially.*

**Proof.**  Let $z={\left(E^{T},I^{T}\right)}^{T}$ and $V\left(z\right)=p^{T}z$. Positivity and $S_{i}\left(t\right)$ not exceeding the envelope used in $\bar{F}$ imply $\dot{z}\leq M\left(t\right)z$. Therefore $\dot{V}\leq p^{T}M\left(t\right)z\leq -\delta p^{T}z=-\delta V$. Gronwall’s inequality yields $V\left(t\right)\leq V\left(T_{0}\right)e^{-\delta (t-T_{0})}$. Since p≫0, every component of *z* is bounded by a positive multiple of *V* and converges exponentially.   □

With a fixed susceptible upper envelope and a polytope containing all admissible comparison matrices, linear programming can verify Equation (14) at every vertex using the same *p* and δ. Sampled-regime checks alone do not certify uniform eradication.

### 2.4. Joint Defense Optimization

Let $w_{i}$ measure damage per infectious device, $c_{i}$ patching cost, and $d_{ij}$ relationship-suppression cost. Define the retained weighted relationship intensity

(15) $$Q_{\mathrm{svc}}\left(v\right)=\frac{\sum_{i,j}w_{ij}^{\mathrm{svc}}a_{ij}^{0}\left(1-v_{ij}\right)}{\sum_{i,j}w_{ij}^{\mathrm{svc}}a_{ij}^{0}},$$

Here $w_{ij}^{\mathrm{svc}}$ measures service importance. The differentiable proxy measures weighted relationship capacity, not connectivity or reachability; known device graphs permit supplementary component, algebraic-connectivity, and service-path checks.

Over horizon [0,T], the defender minimizes

(16) $$\begin{array}{cc} J\left(u,v\right)=\int_{0}^{T}[ & \sum_{i}w_{i}I_{i}+\frac{1}{2}\sum_{i}c_{i}u_{i}^{2}+\frac{1}{2}\sum_{i,j}d_{ij}v_{ij}^{2}\\ \; & +\eta {\left(1-Q_{\mathrm{svc}}\left(v\right)\right)}^{2}]\;dt+\sum_{i}\phi_{i}I_{i}\left(T\right), \end{array}$$

subject to Equations (4)–(7), box limits, and instantaneous capacity constraints

(17) $$\sum_{i}b_{i}u_{i}\left(t\right)\leq B_{u},\;\sum_{i,j}h_{ij}v_{ij}\left(t\right)\leq B_{v}.$$

Weights $b_{i}$ convert per-capita patch hazards to resource use; $h_{ij}$ measures directed-class enforcement cost. The objective penalizes infection burden, action effort, and aggregate service loss, with terminal infection discouraging postponement beyond *T*.

**Proposition** **3** (Existence of an optimal defense)**.** *For finite T, a nonempty compact convex control set, and positive quadratic action costs, problem (16) and (17) admits an optimal control pair.*

**Proof.**  The state is uniformly bounded in Ω. The dynamics are continuous in state and affine in the controls. The admissible-control set is closed, bounded, and convex, and the integrand is convex and coercive in (u,v). A minimizing sequence therefore has a weakly convergent subsequence; continuity of the state map and lower semicontinuity of the integral cost establish existence.   □

Let $\psi_{i}^{S},\psi_{i}^{E},\psi_{i}^{I},\psi_{i}^{R}$ be adjoint variables and let H be the Hamiltonian. The adjoints satisfy

(18) $$\dot{\psi }=-\frac{\partial H}{\partial x},\;\psi_{i}^{S}\left(T\right)=\psi_{i}^{E}\left(T\right)=\psi_{i}^{R}\left(T\right)=0,\;\psi_{i}^{I}\left(T\right)=\phi_{i}.$$

For a fixed state–adjoint pair, define the control-linear coefficients

(19) $$\begin{array}{cc} \ell_{i}^{u}= & \left(\psi_{i}^{R}-\psi_{i}^{S}\right)S_{i}+\left(\psi_{i}^{R}-\psi_{i}^{I}\right)I_{i}, \end{array}$$

(20) $$\begin{array}{cc} \ell_{ij}^{v}= & \left(\psi_{i}^{S}-\psi_{i}^{E}\right)\frac{\beta_{i}S_{i}a_{ij}^{0}I_{j}}{N_{j}}. \end{array}$$

Let $C=\mathrm{diag}(c_{i})$, let $D_{v}=\mathrm{diag}\left(d_{ij}\right)$ after vectorizing the ordered pairs, and set

(21) $$W_{s}=\sum_{kl}w_{kl}^{\mathrm{svc}}a_{kl}^{0},\;\alpha_{ij}=\frac{w_{ij}^{\mathrm{svc}}a_{ij}^{0}}{W_{s}}.$$

Because $1-Q_{\mathrm{svc}}\left(v\right)=\alpha^{T}v$, the control-dependent part of the Hamiltonian, up to terms independent of (u,v), is

(22) $$\frac{1}{2}u^{T}Cu+{\left(\ell^{u}\right)}^{T}u+\frac{1}{2}v^{T}D_{v}v+{\left(\ell^{v}\right)}^{T}v+\eta {\left(\alpha^{T}v\right)}^{2}.$$

Write

$$U=\left\{u:0\leq u_{i}\leq {\bar{u}}_{i},\;b^{T}u\leq B_{u}\right\},\;V=\left\{v:0\leq v_{ij}\leq {\bar{v}}_{ij},\;h^{T}v\leq B_{v}\right\}.$$

The exact constrained patch condition is therefore the *C*-metric projection

(23) $$u{}^{\circ}=-C^{-1}\ell^{u},\;u^{*}=\Pi_{U}^{C}\left(u{}^{\circ}\right):=\arg\min_{u\in U}\frac{1}{2}{\left(u-u{}^{\circ}\right)}^{T}C\left(u-u{}^{\circ}\right).$$

For relation suppression, the service penalty couples all ordered pairs through the positive-definite Hessian

(24) $$H_{v}=D_{v}+2\eta \alpha \alpha^{T}≻0,\;v{}^{\circ}=-H_{v}^{-1}\ell^{v}.$$

With $N_{V}\left(v\right)$ denoting the convex-analysis normal cone, the exact KKT condition and its equivalent metric-projection form are

(25) $$\begin{array}{cc} 0 & \in H_{v}v^{*}+\ell^{v}+N_{V}\left(v^{*}\right),\\ v^{*} & =\Pi_{V}^{H_{v}}\left(v{}^{\circ}\right):=\arg\min_{v\in V}\frac{1}{2}{\left(v-v{}^{\circ}\right)}^{T}H_{v}\left(v-v{}^{\circ}\right). \end{array}$$

Here $N_{V}\left(v\right)=\left\{n:n^{T}\left(y-v\right)\leq 0\;\mathrm{for}\;\mathrm{every}\;y\in V\right\}$, which fixes the normal-cone convention used above. For η>0, the projection metric must retain the rank-one service term. Evaluating these necessary conditions requires forward state and backward adjoint integration with centralized information; infection pressure, structural amplification, action cost, and service value also inform feedback design.

### 2.5. JATP Feedback Policy

Let $c_{i}^{\mathrm{eig}}>0$ be the baseline matrix’s right Perron eigenvector, normalized by $K^{-1}\sum_{i}c_{i}^{\mathrm{eig}}=1$ and recomputed when $A^{0}$ changes. All numerical matrices are symmetric; directed deployments must choose right, left, or sensitivity-based influence consistently with matrix orientation. At epoch $t_{n}$, an external engine supplies ${\hat{T}}_{ij}\left(t_{n}\right)\in \left[0,1\right]$, with higher values indicating stronger trustworthiness evidence. Define latent-infection discounts $\xi_{u},\xi_{v}\in \left[0,1\right]$, patch-score regularizer $\varepsilon =10^{-9}$, trust gain $\alpha_{T}\geq 0$, and independent deficit $\theta_{ij}=1-{\hat{T}}_{ij}$:

(26) $$\begin{array}{cccc} z_{i}^{u} & =c_{i}^{\mathrm{eig}}\left[\hat{I_{i}}+\xi_{u}{\hat{E}}_{i}+\varepsilon \right],\; & z_{i}^{v} & =c_{i}^{\mathrm{eig}}\left[\hat{I_{i}}+\xi_{v}{\hat{E}}_{i}\right],\\ r_{ij} & =z_{i}^{v}+z_{j}^{v},\; & p_{ij} & =r_{ij}\left(1+\alpha_{T}\theta_{ij}\right). \end{array}$$

The dimensionless trust multiplier prioritizes low-trust classes during outbreaks while risk-weighted suppression vanishes with epidemic risk. Trust evidence is separate from prevalence estimates $\hat{E},\hat{I}$.

Let ${\bar{w}}_{\mathrm{svc}}=K^{-2}\sum_{ij}w_{ij}^{\mathrm{svc}}$ and $\delta_{ij}=w_{ij}^{\mathrm{svc}}/{\bar{w}}_{\mathrm{svc}}$ denote the unit-mean service discounts used by the feedback law. These differ from the quadratic objective coefficients $d_{ij}$. With saturation scale τ>0 and the feasible sets U and V defined above, JATP forms

(27) $${\tilde{u}}_{i}=\frac{B_{u}z_{i}^{u}}{b_{i}\sum_{k}z_{k}^{u}},\;{\tilde{v}}_{ij}={\bar{v}}_{ij}\frac{p_{ij}}{p_{ij}+\tau \delta_{ij}},\;u=A_{U}\left(\tilde{u}\right),\;v=\Pi_{V}^{I}\left(\tilde{v}\right),$$

where $A_{U}$ is capped proportional redistribution and $\Pi_{V}^{I}$ is the Euclidean projection onto the box-constrained shared-budget polytope. Capped redistribution is an allocation map, whereas the relationship operator is a metric projection. These two heuristic operators enforce the resource constraints at every control epoch; they differ from the *C*- and $H_{v}$-metric optimality projections in Equations (23) and (25). The service-dependent $\delta_{ij}$ reduces suppression of high-value relationship classes before projection, while $h_{ij}$ represents enforcement resource use. Controls are held until the next decision epoch, and ${\bar{v}}_{ij}<1$ prevents complete removal of an individual relationship class.

JATP is a feasible feedback heuristic motivated by the Perron sensitivity in Equation (12) and the state–adjoint products in Equations (23)–(25). Perron influence approximates long-range amplification, $I_{i}+\xi E_{i}$ approximates near-term source risk, and external $\theta_{ij}$ modulates edge priority. The policy updates actions from measurements without solving a forward–backward boundary-value problem at each control epoch. Its objective value is evaluated separately against an offline numerical benchmark in Section 3.

Each epoch aggregates endpoint and traffic evidence into ${\hat{E}}_{i},{\hat{I}}_{i}$, obtains ${\hat{T}}_{ij}$, forms the scores in Equation (26), and applies capped patch redistribution and projected, service-discounted suppression in Equation (27). Enforcement uses rate limiting, renewed authentication, quarantine, or provider replacement; relationships restore as risk declines.

Score formation costs O(K) and pair-risk formation $O(K^{2})$. A weighted water-filling routine implements the shared-budget Euclidean projection. The online controller uses stratum estimates, a cached K×K relationship summary, epoch-refreshed trust, service weights, and capacities; Perron influence refreshes when the aggregate matrix changes materially. Upstream endpoint or fog collectors process O(n) device reports. Section 3 reports controller computation, upstream aggregation, and command payload separately for K=3–128 and up to $10^{6}$ device records.

Deployment recommendations, not simulated here, are a minimum service-capacity floor for essential classes, hysteresis or minimum hold time against relation flapping, and an emergency service-cost override for high-confidence destructive worms. Section 3 tests the effect of compromised telemetry. Commands should be authenticated, freshness checked, and acknowledged; command-channel Byzantine tolerance remains outside the model.

## 3. Numerical Experiments and Results

### 3.1. Simulation Setup and Evaluation Protocol

The reference configuration comprises ordinary endpoints, gateways, and high-centrality hubs with population shares 0.50, 0.32, and 0.18, respectively. Their transmission coefficients are 0.44, 0.52, and 0.61; activation rates are 0.28, 0.24, and 0.20; native recovery rates are 0.055, 0.070, and 0.090; and immunity-loss rates are 0.006, 0.006, and 0.005. The baseline relationship matrix is

(28) $$A^{0}=\left[\begin{array}{ccc} 0.72 & 0.38 & 0.24\\ 0.38 & 0.92 & 0.48\\ 0.24 & 0.48 & 1.18 \end{array}\right].$$

The external trust evidence and service-importance inputs in the reference experiment are

(29) $$T^{0}=\left[\begin{array}{ccc} 0.92 & 0.78 & 0.68\\ 0.78 & 0.90 & 0.74\\ 0.68 & 0.74 & 0.86 \end{array}\right],\;W^{\mathrm{svc}}=\left[\begin{array}{ccc} 1.00 & 1.10 & 1.25\\ 1.10 & 1.25 & 1.50\\ 1.25 & 1.50 & 1.80 \end{array}\right].$$

Both matrices are synthetic and nonuniform: larger $T_{ij}^{0}$ and $w_{ij}^{\mathrm{svc}}$ indicate stronger trust and greater service importance, respectively. Entries (i,j) represent directed flows from *j* to *i*, so symmetric matrices still require all-entry sums. We set ${\bar{w}}_{\mathrm{svc}}=K^{-2}\sum_{ij}w_{ij}^{\mathrm{svc}}$, $\delta_{ij}=w_{ij}^{\mathrm{svc}}/{\bar{w}}_{\mathrm{svc}}$, $h_{ij}=a_{ij}^{0}/\sum_{kl}a_{kl}^{0}$, and $B_{v}=0.30$, limiting suppression to 30% of normalized baseline directed relationship intensity per epoch. Initial exposed and infected totals are 0.0021 and 0.0040 (stratum values in Appendix C). Over the 120-day horizon, controls are updated every $\Delta_{c}=0.1$ day and held between epochs. Classical fourth-order Runge–Kutta (RK4) integration advances the state; convergence tests vary its internal substep while fixing $\Delta_{c}$.

Time is measured in days. The transition, patch, and repair parameters $\sigma_{i},\gamma_{i},\omega_{i},$ and $u_{i}$ therefore have units day^−1^; population fractions, suppression fractions, trust scores, and scalar contact multipliers are dimensionless. Parameters are selected to exercise heterogeneous dynamics rather than fit a named malware family. The numerical study therefore evaluates mechanisms and relative policy robustness; the implications for external validity are discussed in Section 4.5.

The five policies are no control, static patching ($u_{i}=0.045$), risk-only patching without relation control, topology-only control, and JATP. With $b_{i}=N_{i}$, static and adaptive patching share $B_{u}=0.045$ day^−1^. Metrics are peak infected fraction and time, burden $E_{I}=\int_{0}^{T}\sum_{i}I_{i}\left(t\right)\;dt$, final infected fraction, patch effort $E_{u}=\int_{0}^{T}\sum_{i}b_{i}u_{i}{\left(t\right)}^{2}\;dt$, topology effort $E_{v}=\int_{0}^{T}\sum_{ij}\delta_{ij}v_{ij}{\left(t\right)}^{2}\;dt/\sum_{ij}\delta_{ij}$, and time-averaged retained relationship intensity

(30) $${\bar{Q}}_{\mathrm{svc}}=T^{-1}\int_{0}^{T}Q_{\mathrm{svc}}\left(v\left(t\right)\right)\;dt.$$

The three intervention metrics use the applied zero-order-held commands, including under observation delay and noise. The service-weight ablation evaluates both histories with fixed reference costs $\delta_{ij}^{\mathrm{ref}}=w_{ij}^{\mathrm{svc}}/{\bar{w}}_{\mathrm{svc}}$, giving the common quadratic metric $E_{v}^{\mathrm{ref}}$.

Risk-only patching is the primary comparator because it uses the same infection-aware patch allocation as JATP. Static patching reveals the value of state-dependent allocation. Topology-only control tests whether relation intervention can substitute for patching. No control provides the natural epidemic reference.

Because patch production and relationship enforcement are distinct resources, the reference comparison uses separate capacities. An additional comparison imposes a common dimensionless expenditure

(31) $$C_{\mathrm{int}}=\frac{1}{T}\int_{0}^{T}\left[\sum_{i}N_{i}{\left(\frac{u_{i}}{{\bar{u}}_{i}}\right)}^{2}+\sum_{ij}h_{ij}{\left(\frac{v_{ij}}{{\bar{v}}_{ij}}\right)}^{2}\right]\;dt.$$

Equation (31) uses enforcement weights $h_{ij}$ rather than service-cost weights $\delta_{ij}$. The common ceiling $C_{\mathrm{int}}\leq C_{\mathrm{int}}^{\mathrm{JATP}}=0.1963840574$ preserves $B_{u}=0.045$, $B_{v}=0.30$, ${\bar{u}}_{i}=0.15$, and ${\bar{v}}_{ij}=0.72$. Risk-only is unchanged; topology-only meets the ceiling by increasing its saturation parameter to 0.05193486 through bisection. Permitted resources are equal, but expenditure need not be: unused expenditure cannot increase instantaneous patch capacity.

The optimization benchmark minimizes the complete objective in Equation (16), with the fixed weights

(32) $$w_{i}=1,\;c_{i}=\frac{2N_{i}}{T{\bar{u}}_{i}^{2}},\;d_{ij}=\frac{2\delta_{ij}}{T{\bar{v}}_{ij}^{\;2}\sum_{kl}\delta_{kl}},\;\eta =\frac{1}{T},\;\phi_{i}=12.$$

These fixed weights evaluate every policy using time-averaged intervention and squared service-loss penalties, infection burden, and terminal infection. The topology weights in $C_{\mathrm{int}}$ and *J* distinguish enforcement usage from service cost.

Direct single shooting uses three patch and nine directed suppression commands over twelve 10-day blocks (144 variables). Sequential least-squares programming (SLSQP) uses differentiated RK4 sensitivities, a 0.5-day step, tolerance $2\times 10^{-9}$, and at most 250 iterations. Starts are 10-day JATP averages and uniform feasible commands; the better feasible result initializes 24 five-day blocks (288 variables). Schedules are re-evaluated at a 0.1-day step, checking box limits, instantaneous capacities, cumulative expenditure, positivity, and population balance. This yields local solutions of the discretized expenditure-constrained problem, not a global optimum or receding-horizon control.

Trust quality is tested against prevalence-only topology control: JATP with $\alpha_{T}=0$, retaining its other laws, capacities, and service weights. Unlike risk-only patching, this comparator controls relationships. The experiment is specified independently of simulated infection outcomes. With $H_{\min}$ and $H_{\max}$ denoting the extrema of $H_{ij}$ over ordered classes in the nonconstant reference matrix, nominal topology defines

(33) $$H_{ij}=a_{ij}^{0}\left(\beta_{i}c_{i}^{\mathrm{eig}}+\beta_{j}c_{j}^{\mathrm{eig}}\right),\;T_{ij}^{★}=0.95-0.85\frac{H_{ij}-H_{\min}}{H_{\max}-H_{\min}},$$

Lower $T_{ij}^{★}$ denotes greater structural propagation potential. Only this experiment adds residual channel vulnerability by multiplying edge-class (i,j) transmission by

(34) $$g_{ij}^{★}=\frac{1+\kappa_{T}\left(1-T_{ij}^{★}\right)}{\sum_{kl}h_{kl}\left[1+\kappa_{T}\left(1-T_{kl}^{★}\right)\right]},\;\kappa_{T}=2.$$

Normalization fixes mean contact opportunity, $\sum_{ij}h_{ij}g_{ij}^{★}=1$. Evidence quality varies through

(35) $${\hat{T}}_{ij}\left(\chi_{T}\right)={\mathrm{clip}}_{\left[0.05,0.95\right]}\left\{{\bar{T}}_{h}+\chi_{T}\left(T_{ij}^{★}-{\bar{T}}_{h}\right)\right\},\;{\bar{T}}_{h}=\sum_{ij}h_{ij}T_{ij}^{★},$$

for $\chi_{T}=-1,-0.75,\ldots ,1$: $\chi_{T}=1$ preserves the informative ordering, $\chi_{T}=0$ removes it, and $\chi_{T}<0$ reverses it. At each level, bisection adjusts only τ to match realized $E_{v}$ to prevalence-only control in the same latent-risk system, preserving boxes and the instantaneous budget. Model-derived $T^{★}$ tests a conditional allocation mechanism at matched effort, not empirical trust validity.

Explicit-network validation uses individual SEIR probabilities on sparse three-block graphs (target mean degree 18), with independent lognormal vulnerability multipliers of log-standard deviation 0.20. Infected-neighbor sums from each source block are normalized by the target block’s mean neighbor count in that source block, preserving degree differences while matching $A^{0}$; absent edges contribute zero. JATP and risk-only share twelve graph seeds per n=100,300,1000. Temporal tests independently regenerate relationships every five days; churn also replaces 5% of devices with susceptible entrants while fixing size and stratum shares. A 1000-node stress test adds lognormal activity variation of log-standard deviation 0.8. These deterministic individual-based mean-field simulations use 0.05-day Euler steps and 0.1-day control holds, with matched 0.1-day-step checks; they are not stochastic infection or field experiments.

The robustness matrix in Table 3 summarizes the expanded experiment set. Paired Monte Carlo sampling applies the same uncertain parameters to risk-only and JATP, reducing comparison variance. Transmission, recovery, and activation multipliers follow $\log N(0,s^{2})$ with log-standard deviations 0.15, 0.12, and 0.10, respectively; they therefore have a median of one. Edge multipliers use log-standard deviation 0.12, after which each off-diagonal pair is averaged to obtain a symmetric perturbation; that average is positive but is not itself exactly lognormal. Immunity loss is held fixed in this experiment and varied separately in the local-sensitivity analysis. Observation noise is multiplicative, independently generated by stratum and control epoch, and clipped to the multiplier range 0.05–3. All pseudo-random generators use recorded seeds. All simulations preserve the population of each stratum up to numerical tolerance.

### 3.2. Baseline Efficacy, Service Cost, and Adaptive Allocation (RQ1–RQ2)

Table 4 reports baseline outcomes for the five policies. Without control, infection peaks at 0.5158 on day 25.5 and cumulative burden reaches 19.5095. Static and risk-only patching reduce the peak to 0.1344 and 0.1273, respectively. Risk-aware allocation therefore improves both the peak and cumulative burden in the three-stratum reference system.

Topology-only control peaks at 0.4458 and achieves ${\bar{Q}}_{\mathrm{svc}}=0.7333$. Suppression prevents exposures but cannot repair sources already infected. JATP achieves the lowest peak, 0.0644, and lowest cumulative burden, 2.8717. Relative to risk-only patching, peak and burden fall by 49.43% and 38.97%. JATP achieves ${\bar{Q}}_{\mathrm{svc}}=0.8184$. Patch effort is 0.2503 for JATP and 0.2490 for risk-only, a difference below 0.6%. The shared topology constraint is active during 35.17% of JATP control intervals, compared with 93.83% under topology-only control, confirming that $B_{v}$ is operational rather than nominal.

Under the common ceiling (Table 5), risk-only spends $C_{\mathrm{int}}=0.092224$; the other policies use approximately 0.196384. JATP’s J=3.15064 is 59.56% above the best feasible numerical result, J=1.97461 for five-day blocks. This relative objective excess is not a certified global optimality gap. The numerical schedule improves peak, burden, and retained intensity but ends with higher infection (0.00651 versus 0.00253), reflecting the finite-horizon trade-off in *J*.

Both 10-day starts converge in 29 iterations with an objective difference below $8\times 10^{-10}$; the five-day refinement takes 31 iterations. Recorded solve times are 4.75, 4.82, and 5.91 s, excluding initialization and verification; these are not worst-case bounds. Refining RK4 from 0.5 to 0.1 day changes *J* by at most $5.48\times 10^{-5}\%$, and independent adaptive integration agrees within relative error $7.6\times 10^{-10}$. The maximum constraint residual is $2.21\times 10^{-10}$ (tolerance $10^{-7}$); states remain nonnegative and population residuals below $8\times 10^{-16}$.

**Figure 1:**
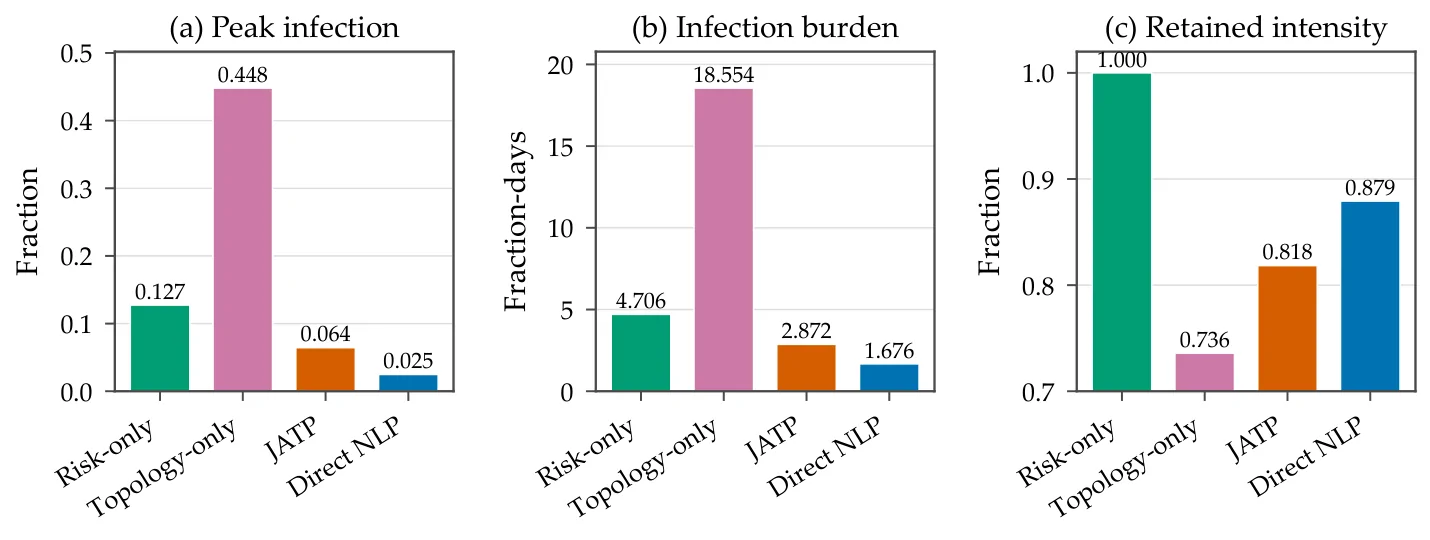
Comparison under the common expenditure ceiling and unchanged instantaneous capacities. Risk-only uses less than the ceiling; direct NLP denotes the locally optimized 5-day open-loop schedule using the full objective.

Figure 1 contrasts nominal-model offline schedules with measurement-based JATP feedback. The dimensionless ceiling is not a calibrated monetary cost.

**Figure 2:**
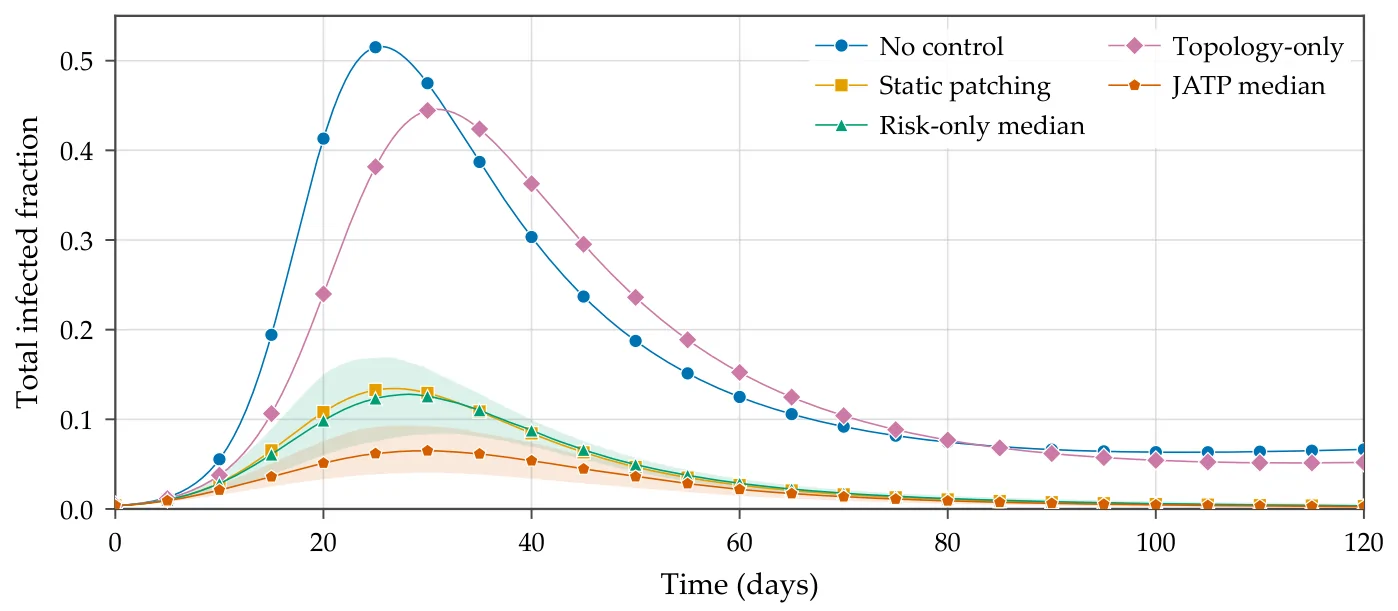
Infection trajectories under five policies. For risk-only patching and JATP, solid centerlines are pointwise medians and shaded envelopes are pointwise 5th–95th percentiles over 200 paired parameter realizations; the other policies show reference trajectories. Shape markers distinguish the policies and are displayed every five days.

Figure 2 shows the timing mechanism and its parameter uncertainty. Relation suppression immediately reduces exposure creation; targeted patching then removes the sources that remain. The JATP median trajectory avoids the high-prevalence regime and remains lower through the late horizon; the pointwise uncertainty envelope visualizes how this advantage persists beyond a single parameter vector.

**Figure 3:**
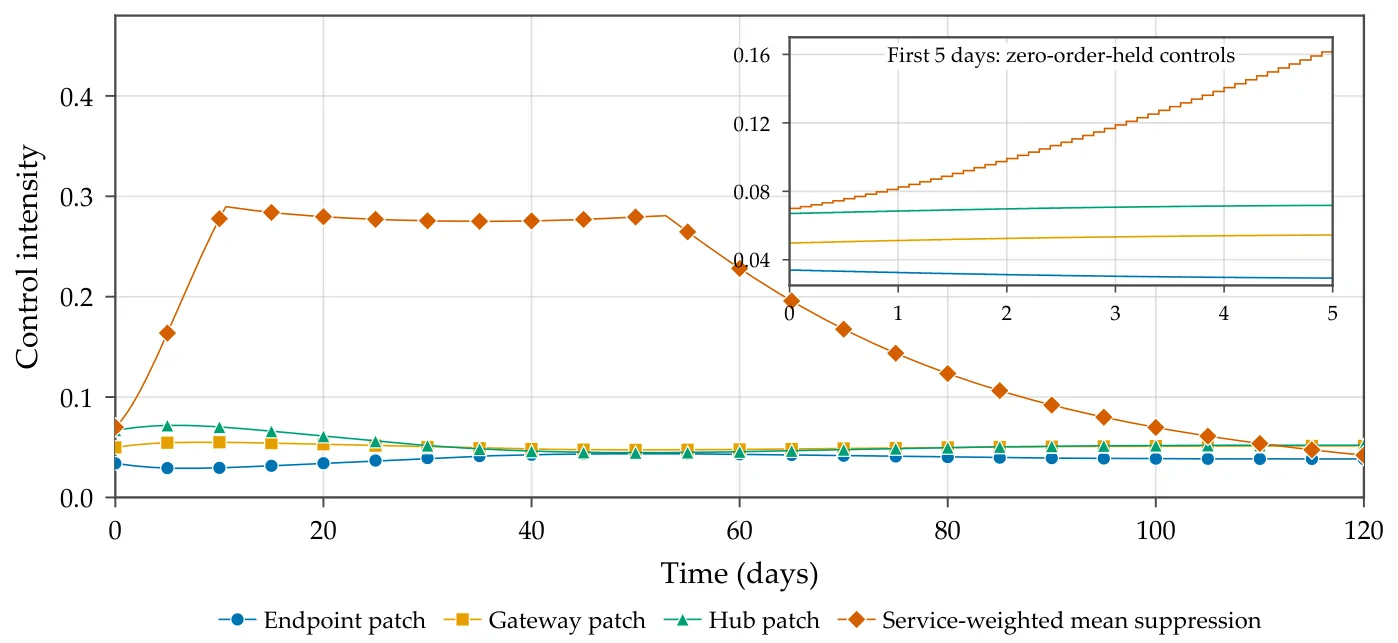
Sampled-data JATP controls. The red service-weighted mean-suppression curve changes slope near days 10.7 and 52.9 when the shared topology budget activates and releases; differing display and budget weights explain why it is not flat while binding. Full-panel markers occur every five days. The unmarked inset shows the actual 0.1-day zero-order-held commands over the first five days, whereas the full panel connects samples for readability.

To explain how the aggregate gain is produced, Figure 3 displays JATP control trajectories. At the first control epoch, population-weighted allocation and Perron influence give the smaller but more influential hub and gateway strata higher per-capita patch rates while preserving the common budget $\sum_{i}N_{i}u_{i}=0.045$. The allocation then adapts with the estimated exposed and infected fractions. Service-weighted mean suppression rises during epidemic growth and relaxes as risk declines. The underlying topology budget binds from approximately day 10.7 through day 52.8; because display and resource weights differ, the red curve can vary slightly during this interval. The inset shows the actual zero-order-held commands during the first five days, whereas the full panel connects samples for readability. Automatic restoration avoids paying service cost as estimated epidemic risk declines.

### 3.3. Budget-Feasible Initial-State Threshold Crossing (RQ3)

At contact scale 0.18, Table 6 evaluates $R_{\mathrm{inst}}\left(N;u,v\right)$ under the simulation capacities: $\sum_{i}N_{i}u_{i}=u\leq B_{u}=0.045$ and $\sum_{ij}h_{ij}v_{ij}=v\leq B_{v}=0.30$. Neither channel alone brings the index below one; their maximum feasible combination gives 0.9798. Contact suppression and shortened infectious duration therefore combine multiplicatively to cross this outbreak-onset threshold. The calculation uses fully susceptible S=N, unlike the constant-control MFE threshold $R_{0}$, which uses $S^{*}\left(u\right)$.

**Figure 4:**
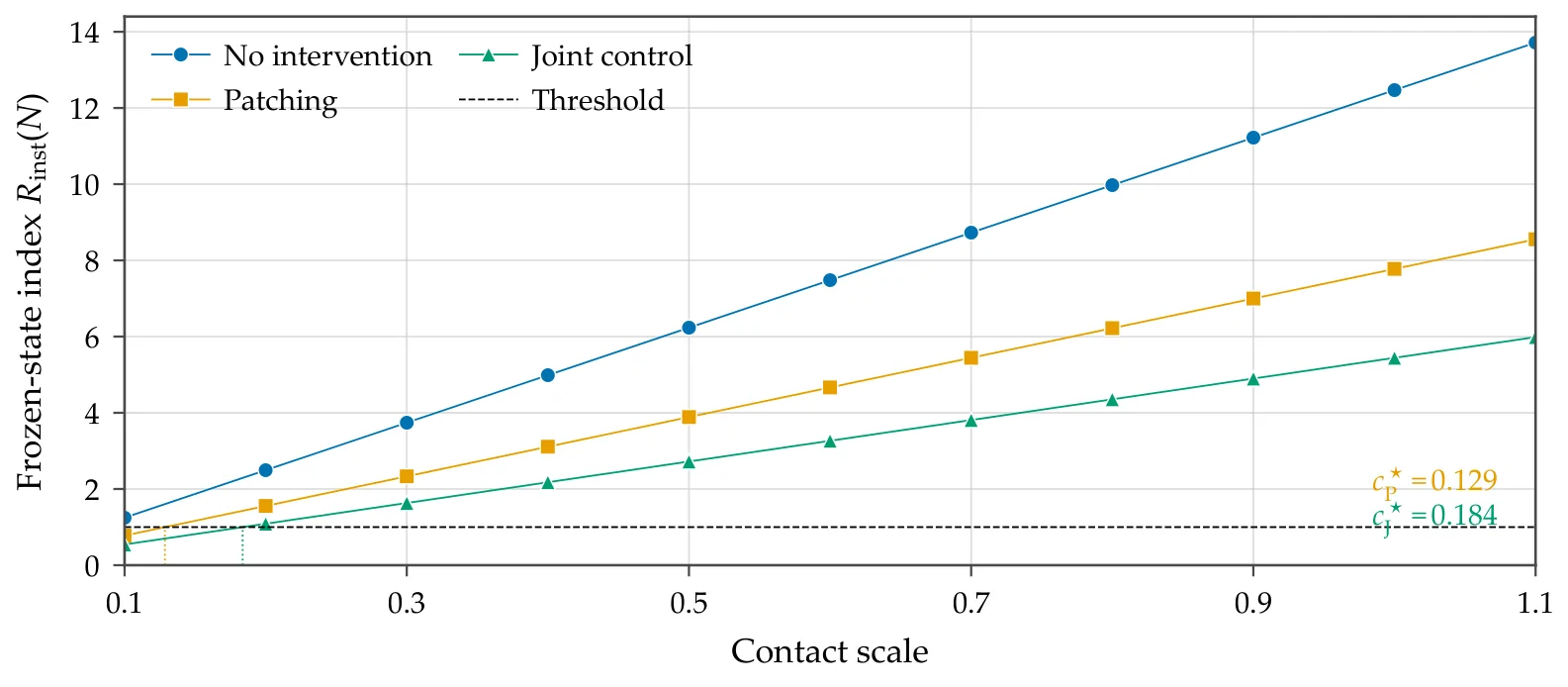
Fully susceptible frozen-state reproduction index versus the scalar contact multiplier. Patching uses the feasible uniform intensity u=0.045; joint control additionally applies the feasible uniform suppression v=0.30. Shape markers are displayed at contact-scale intervals of 0.1. Thin vertical guides mark the in-range $R_{\mathrm{inst}}=1$ crossings; the no-intervention crossing $c^{★}=0.080$ lies to the left of the displayed interval.

Figure 4 extends the initial-state calculation across contact scale *c*. Writing $R_{\mathrm{inst}}^{\left(c\right)}$ for the index obtained after replacing $A^{0}$ by $cA^{0}$, linearity of the frozen-state operator gives $R_{\mathrm{inst}}^{\left(c\right)}\left(N;u,v\right)=cR_{\mathrm{inst}}^{\left(1\right)}\left(N;u,v\right)$. The critical scales are 0.080 without intervention, 0.129 with maximum feasible uniform patching, and 0.184 under the feasible joint action. The uncontrolled crossing lies to the left of the displayed interval; the two in-range crossings are marked by thin guides. Joint control therefore raises the admissible contact scale by a factor of 1.43 relative to patching alone.

### 3.4. Resource, Contact, Topology, and Trust-Evidence Robustness (RQ4)

Increasing the patch-budget scale from 0.50 to 1.50 lowers JATP peak infection from 0.1900 to 0.0270 and cumulative burden from 8.2612 to 1.0166 (Table 7). The return is nonlinear. Moving from 0.50 to 0.75 reduces peak infection by 41.3%, while moving from 1.25 to 1.50 reduces it by 31.8% from a much smaller base. The mean retained weighted relationship intensity rises from 0.7511 to 0.8948 because rapid source removal reduces the need for relation suppression. Consistently, the fraction of intervals at the shared topology budget falls from 59.75% to 1.83%. Thus patch resources buy both security and retained relationship intensity.

**Figure 5:**
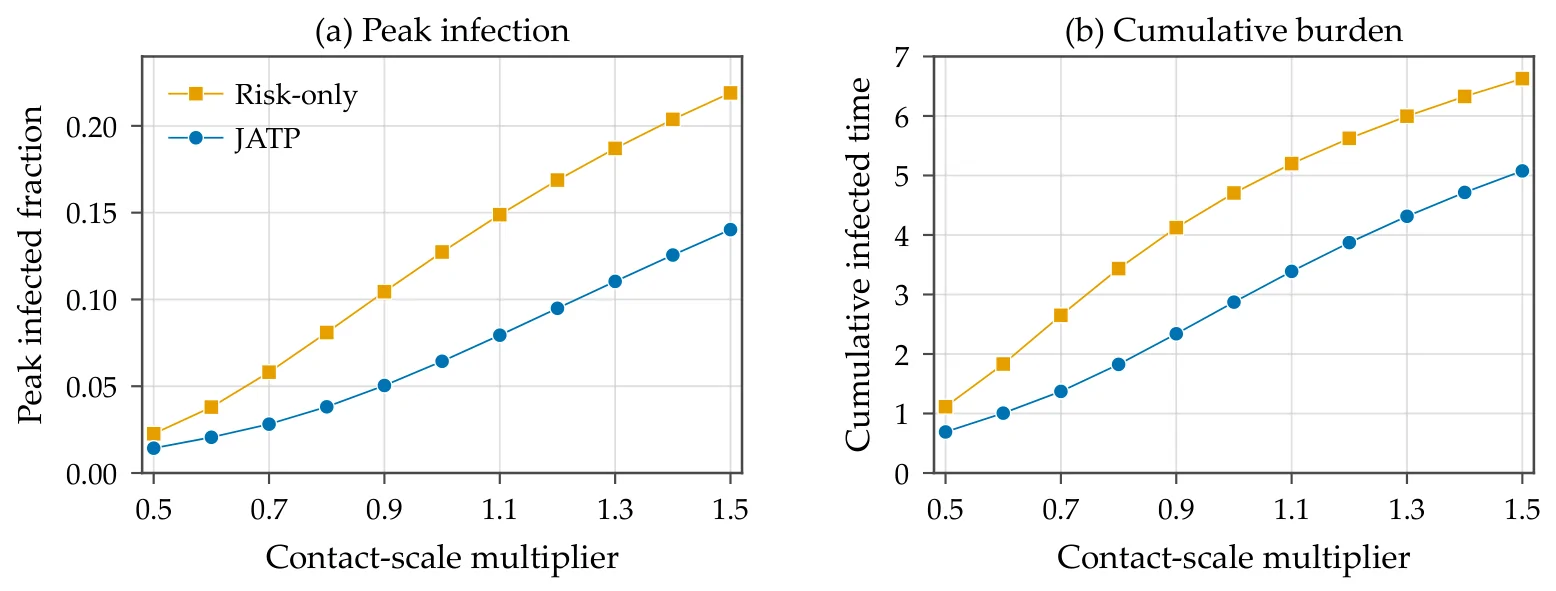
Robustness to the scalar contact multiplier across 11 simulated stress levels from 0.50 to 1.50. The separation between JATP and risk-only patching persists for both peak infection and cumulative burden. Every marker denotes a separately simulated setting; connecting segments only guide the eye.

Increasing contact intensity without changing patch capacity raises infection under both policies (Figure 5). Across 11 scales from 0.50 to 1.50, JATP reduces peak and burden by 35.96–52.86% and 23.40–48.24%, respectively, relative to risk-only patching.

**Figure 6:**
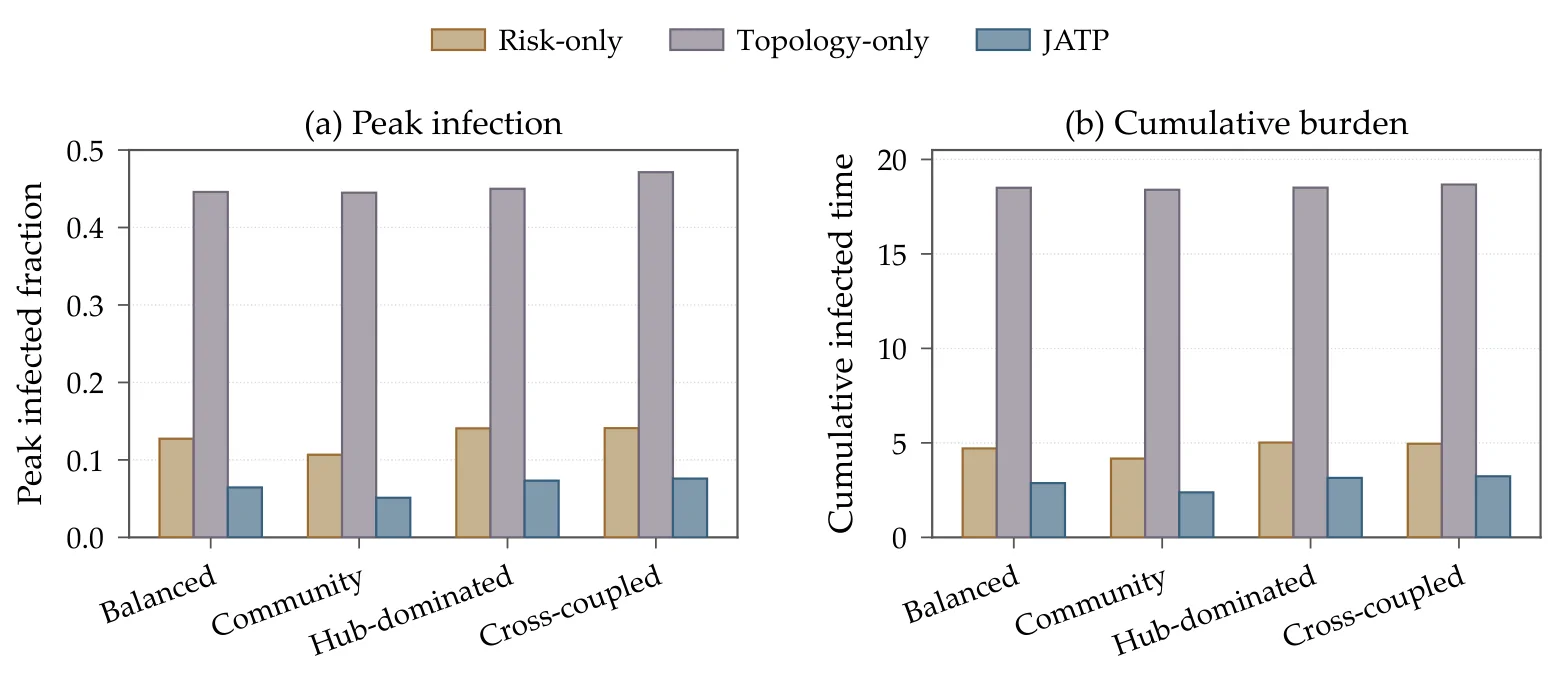
Policy outcomes across four synthetic baseline topology matrices. The corresponding spectral radii are 1.727 (balanced), 1.460 (community), 2.071 (hub-dominated), and 1.758 (cross-coupled). Bars denote separate deterministic simulations for each categorical topology; JATP yields the lowest peak and cumulative burden in every matrix, whereas topology-only control remains insufficient.

JATP gives the lowest peak and burden across balanced, community, hub-dominated, and cross-coupled matrices (Figure 6). Relative to risk-only, peak reductions are 49.4%, 52.1%, 48.0%, and 46.2%, and burden reductions are 39.0%, 42.9%, 37.3%, and 34.8%. The cross-coupled case has the largest controlled outcomes despite the hub-dominated matrix’s larger spectral radius. Perron influence is recomputed for each matrix, allowing the structural control ranking to change.

The mean retained weighted relationship intensity under JATP ranges from 0.8060 in the cross-coupled network to 0.8242 in the community network. The higher retained intensity in the community matrix is consistent with weaker bridges limiting global amplification, although the aggregate metric does not isolate edge-level causal contributions. Topology-only control remains poor across all tested matrices, reinforcing the conclusion that relation intervention is complementary rather than sufficient.

**Figure 7:**
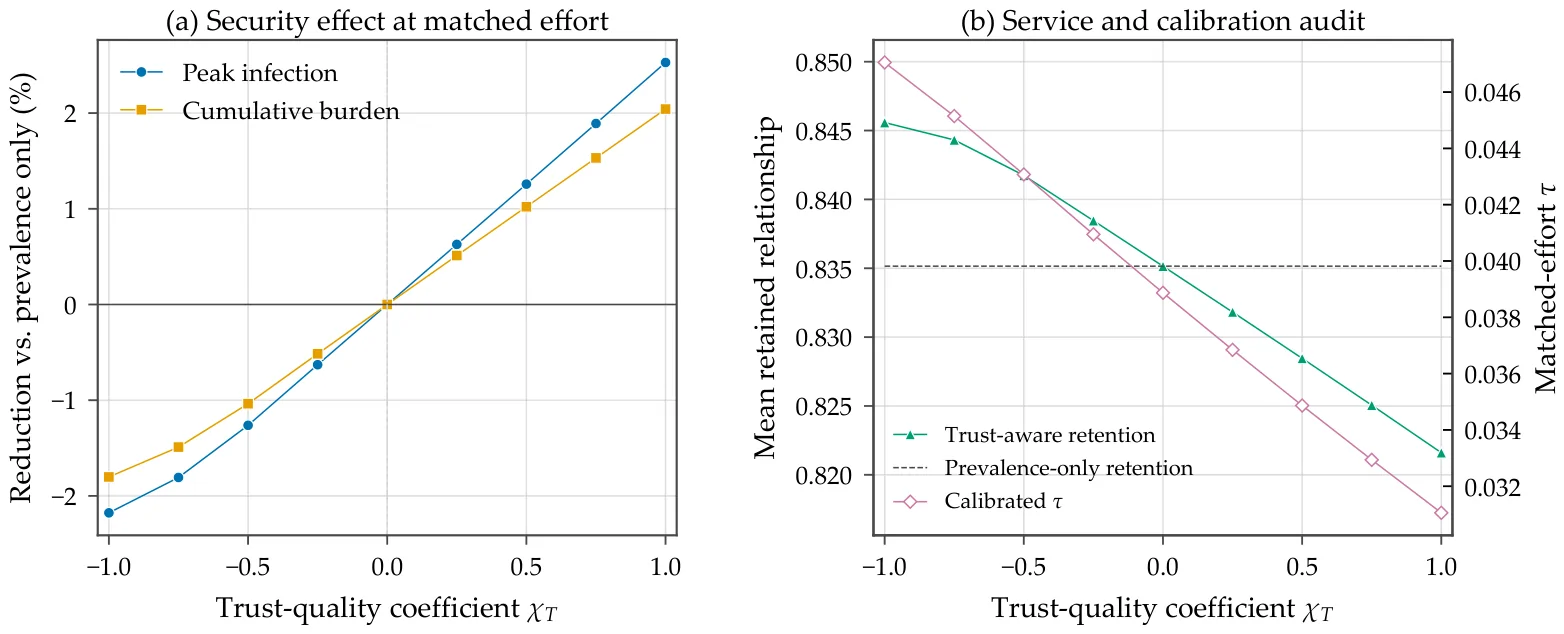
Trust-evidence quality in the latent-edge-risk experiment. Panel (**a**) reports infection reduction relative to prevalence-only control after separately matching realized quadratic topology effort at every quality level. Positive $\chi_{T}$ aligns evidence with the model-defined structural edge risk; $\chi_{T}=0$ removes edge ordering. Panel (**b**) reports retained weighted relationship intensity and the calibrated saturation parameter, making the associated service reallocation explicit. Every marker is a separate deterministic simulation and the connecting lines only guide the eye.

At $\chi_{T}=0$, calibration absorbs the uniform trust multiplier, matching prevalence-only peak and burden within $1.2\times 10^{-4}\%$ (Figure 7). Outcomes improve monotonically to $\chi_{T}=1$, where peak falls from 0.053264 to 0.051917 (2.53%) and burden from 2.40774 to 2.35857 (2.04%); reversing evidence reverses the effect. Effort mismatch stays below 0.0006% across all nine calibrations. Across four latent-risk amplitudes $\kappa_{T}=0.5$–2.0, matched-effort peak reductions increase from 1.40% to 2.53% and burden reductions from 1.10% to 2.04%.

This gain costs retained relationship intensity: it falls from 0.83515 under prevalence-only control to 0.82161 at $\chi_{T}=1$ as matched effort shifts between classes. Panel (b) reports this trade-off and calibrated τ.

**Figure 8:**
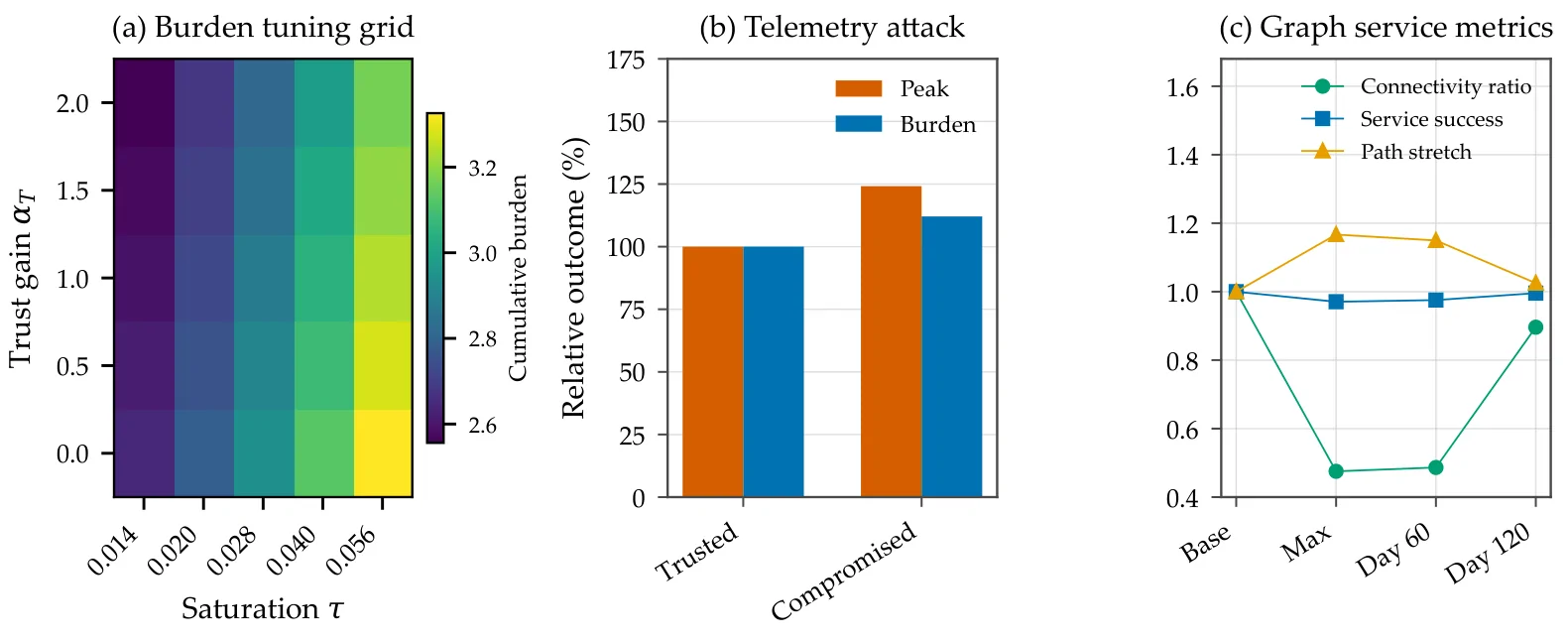
Controller sensitivity, adversarial telemetry, and graph-level service performance. (**a**) Infection burden over the fixed 5×5 controller-parameter grid. (**b**) Peak and burden under trusted and compromised telemetry, normalized to the trusted reference. (**c**) Graph-level service metrics at baseline, maximum suppression, day 60, and day 120; connecting segments only guide the eye between separately evaluated snapshots.

On the Cartesian grid $\alpha_{T}\in \left\{0,0.5,1,1.5,2\right\}$ and τ∈{0.014,0.020,0.028,0.040,0.056} (Figure 8a), peak spans 0.05627–0.07664, burden 2.55608–3.32602, retained intensity 0.77970–0.86473, and normalized cost 0.15789–0.23677. Smaller τ and larger $\alpha_{T}$ generally exchange more effort and service loss for security. The reference $\left(\alpha_{T},\tau \right)=\left(1,0.028\right)$ is median on all four measures; no point improves infection, retention, and effort simultaneously.

Reference service weights reduce peak by 3.42% and burden by 3.07%, and raise reference-weighted ${\bar{Q}}_{\mathrm{svc}}$ by 0.53 percentage points, at 2.90% greater reference-weighted topology effort (Table 8).

On a 300-node reference graph, JATP suppression defines independently sampled edge-retention snapshots at maximum suppression, day 60, and day 120 (30 realizations each). For an undirected edge between classes *i* and *j*, retention probability is $1-(v_{ij}+v_{ji})/2$. Five hundred baseline-connected pairs define path stretch and success, with success requiring a path no longer than 1.5 times its baseline length. All snapshots remain connected, but maximum suppression reduces algebraic connectivity to 0.4754 of baseline, increases mean path stretch to 1.1669, and lowers success to 97.07% (Figure 8c). These topological proxies reveal lost redundancy and routing efficiency rather than measured application performance.

### 3.5. Observation and Parameter Robustness (RQ5)

Feedback policies may receive delayed telemetry. Table 9 reports selected results at 20% multiplicative observation noise. With current observations the mean peak is 0.0646. Delays of one, two, four, and six days increase it to 0.0666, 0.0687, 0.0732, and 0.0780. Even the six-day delayed/noisy case remains far below the risk-only reference peak of 0.1273. Cumulative burden rises from 2.8798 to 3.2441, while the mean retained weighted relationship intensity stays in the narrow range 0.8189–0.8196. The extended delay range represents a prolonged telemetry outage rather than routine feedback latency.

**Figure 9:**
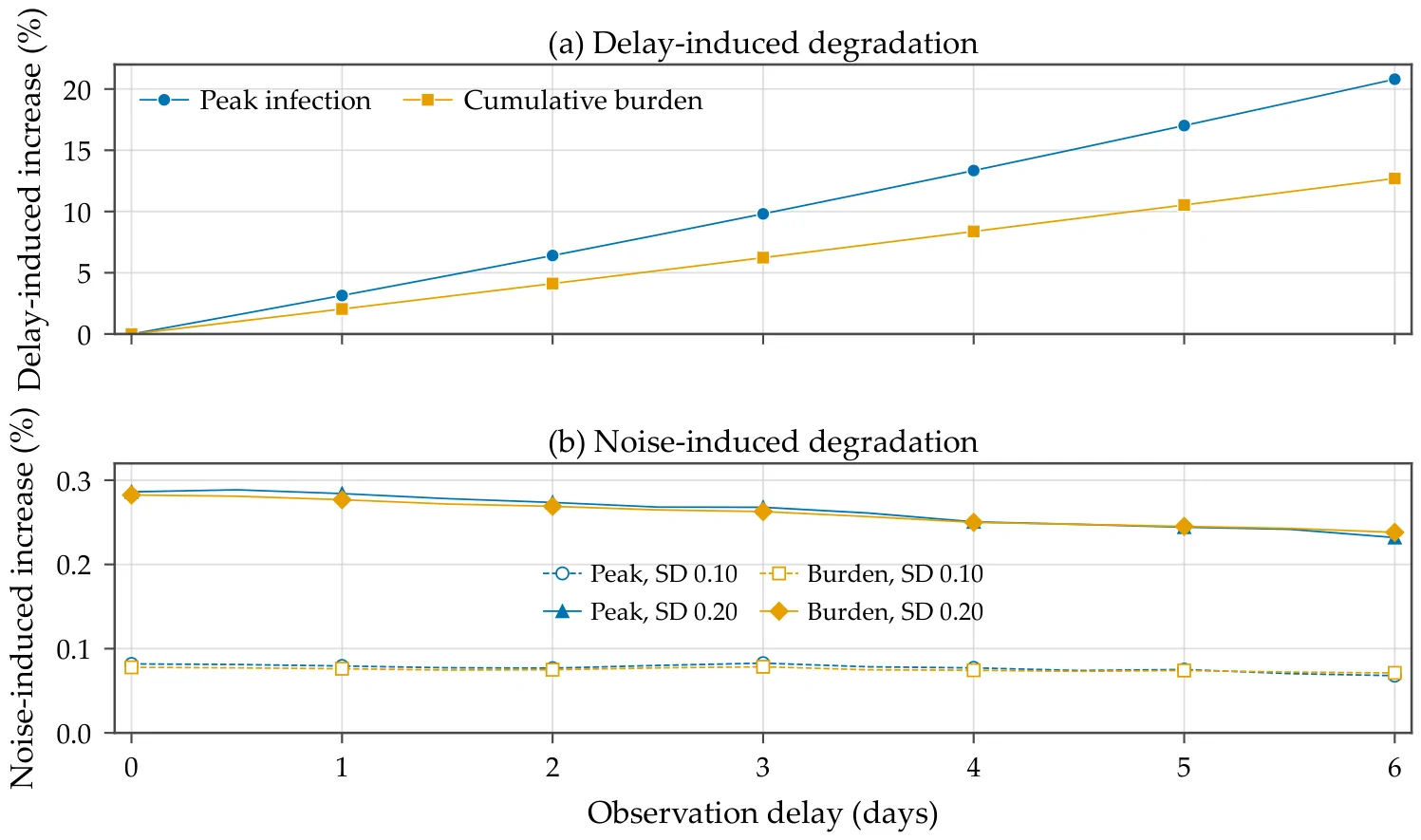
Observation robustness over 13 delays and three noise levels (10 fixed-seed replications per setting; 390 runs). Panel (**a**) shows noise-free increases relative to zero delay; panel (**b**) shows noise-induced increases relative to the no-noise mean at each delay. Color distinguishes outcomes, and dashed/open versus solid/filled curves denote noise SDs 0.10 versus 0.20. Markers occur every day; Table 9 reports absolute outcomes.

Figure 9 separates delay-induced and noise-induced degradation on common percentage scales. Panel (a) reports $\delta_{d}^{q}\left(d\right)=100\left[\bar{q}\left(d,0\right)-\bar{q}\left(0,0\right)\right]/\bar{q}\left(0,0\right)$ for the noise-free peak and burden, while panel (b) reports $\delta_{\sigma }^{q}\left(d,\sigma \right)=100\left[\bar{q}\left(d,\sigma \right)-\bar{q}\left(d,0\right)\right]/\bar{q}\left(d,0\right)$ at the same delay. Across the tested delays, 10% noise increases mean peak infection and cumulative burden by only 0.068–0.083% and 0.071–0.079%, respectively; the corresponding ranges under 20% noise are 0.232–0.289% and 0.238–0.283%. By comparison, extending the noise-free delay from zero to six days increases peak infection by 20.80% and cumulative burden by 12.70%, confirming that stale-state information dominates the tested independent noise.

Coordinated bias is more damaging: during days 5–50, one compromised feed reports 20% of the hub state and inflates endpoint state by 80%. It raises peak from 0.06439 to 0.07995 (24.16%) and burden from 2.87173 to 3.21831 (12.07%; Figure 8b). This sensitivity motivates telemetry-integrity safeguards [40,42].

**Figure 10:**
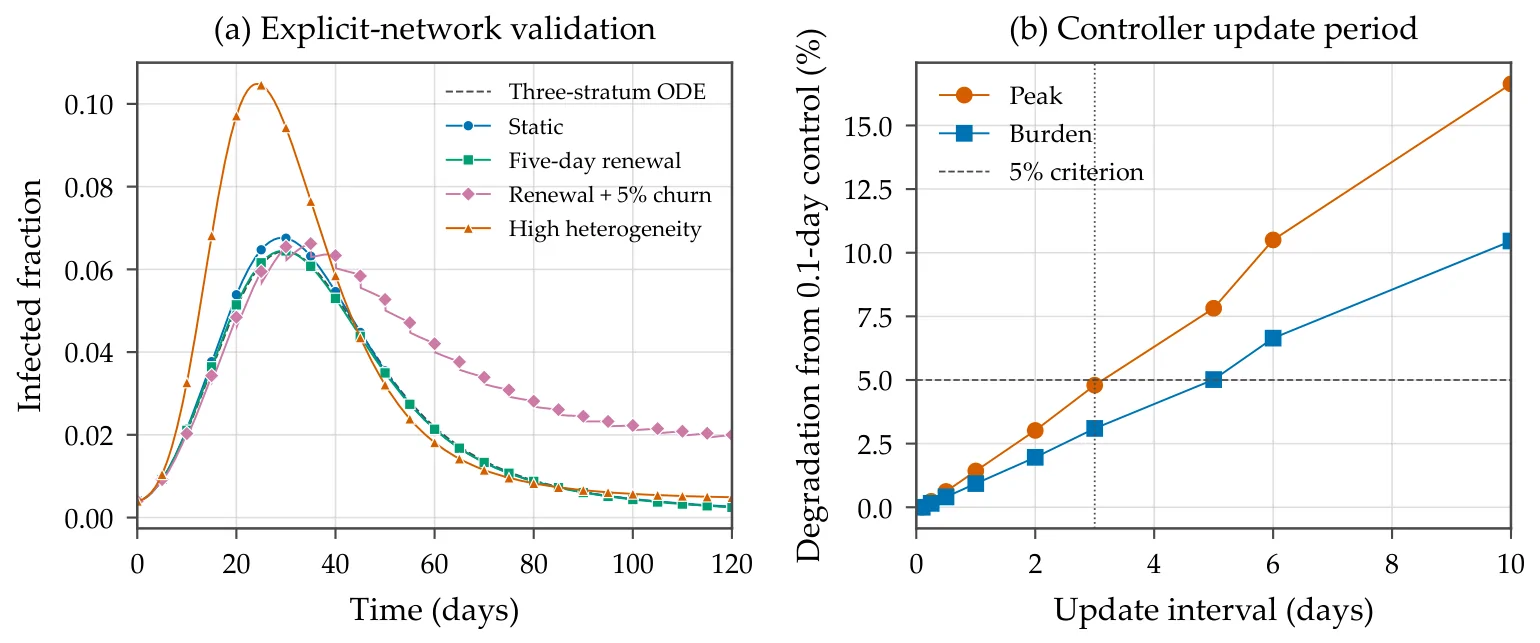
Tests beyond the aggregate reference. (**a**) Mean trajectories over 12 realizations at n=1000: renewal remains near the ODE, susceptible-entry churn raises the tail, and strong activity heterogeneity raises the peak. (**b**) Degradation with controller interval; the vertical line identifies the largest tested interval meeting the illustrative 5% criterion for both outcomes.

With integration accuracy fixed, longer zero-order-held controller intervals worsen performance (Figure 10b, 0.1–10-day sweep). Relative to 0.1-day updates, three-day decisions increase peak by 4.80% and burden by 3.10%; five-day decisions increase them by 7.82% and 5.02%. Three days is the largest tested interval meeting an illustrative 5% tolerance for both outcomes, not a general deployment bound; finer intermediate intervals were not tested.

Table 10 summarizes 200 paired realizations of transmission, activation, recovery, and relationship parameters, also used in Figure 2. JATP lowers mean peak and burden while retaining mean ${\bar{Q}}_{\mathrm{svc}}=0.8189$ (5th percentile 0.7992).

Paired mean and median differences, parametric confidence intervals, and standardized effects are reported in Table 11. Mean bootstrap 95% CIs are 0.06081–0.06339 for peak and 1.77825–1.81420 for burden, using 10,000 paired resamples (seed 20260916). Both paired *t* and one-sided Wilcoxon signed-rank tests reject zero improvement ($p<10^{-34}$).

Mean relative reductions are 48.83% (bootstrap 95% CI 48.44–49.21%) for peak and 38.64% (38.03–39.24%) for burden. These intervals apply to the specified paired parameter-uncertainty distribution, with immunity loss held fixed.

Finally, centered ±10% perturbations yield the dimensionless local elasticity $E_{Y}=\left[Y\left(1.1\theta \right)-Y\left(0.9\theta \right)\right]/\left[0.2Y\left(\theta \right)\right]$, varying one parameter group at a time. Peak infection is most sensitive to transmission/contact (2.251), followed by recovery (−1.012), activation (0.745), and immunity loss (0.113). Cumulative burden has elasticities 1.826, −1.192, 0.392, and 0.347, respectively. The equality of transmission and contact elasticities follows from their multiplicative appearance in Equation (3) when every transmission coefficient or every contact is scaled uniformly.

The results have operational meaning. Rapidly reducing contact opportunity is valuable for peak control, whereas durable recovery is especially important for total burden. Immunity loss has little effect on the first peak but a larger effect on the tail. These distinct sensitivities justify reporting peak infection and cumulative burden as complementary evaluation outcomes; only running and terminal infection enter the finite-horizon objective in Equation (16).

### 3.6. Explicit-Network and Scalability Validation (RQ6)

With moderate heterogeneity and matched block mixing, explicit-network JATP trajectories remain close to the ODE (Table 12). Paired peak reductions relative to node-level risk-only are 47.53–47.67% for static and 48.84–48.90% for renewed networks; burden reductions are 37.20–37.37% and 38.50–38.75%, respectively.

Susceptible-entry churn adds a flow absent from the closed ODE: replacing 5% of devices every five days raises JATP burden by 46.18–49.68% relative to that prediction, although burden remains 43.81–44.77% below risk-only under identical turnover.

At n=1000, activity heterogeneity raises the degree coefficient of variation from 0.239 to 0.944. JATP peak and burden exceed aggregate predictions by 62.92% and 30.47%, with 25.44% NRMSE; paired gains over risk-only shrink to 34.12% and 26.10%. This mismatch motivates testing finer degree or community strata (Figure 10a).

The complete controller is benchmarked on an AMD Ryzen 7 6800H (nine batches of twenty epochs). With an active topology budget, median times at K=3,32,128 are 0.506, 0.656, and 2.457 ms, including risk scoring, capped patch redistribution, and 80 projection iterations. Perron refresh, needed only after aggregate-topology changes, takes 0.034, 0.205, and 7.293 ms. At K=128, two incoming state vectors, refreshed relationship/trust matrices, and outgoing patch/relationship commands total $3K^{2}+3K$ 32-bit values: 193.5 KiB per epoch, or 129.5/65.5 KiB with topology/both matrices cached. Full-payload serialization is estimated at 0.159 s over 10 Mbit/s. Vectorized in-memory aggregation of two states for $10^{6}$ devices into 32 strata takes median 2.42 ms. These measurements cover local computation, not end-to-end latency. Trust inference, telemetry acquisition, authentication, headers, retransmission, propagation, and actuation require separate measurement.

### 3.7. Numerical Convergence

With controller updates fixed at 0.1 day, RK4 step refinement gives errors far below the policy differences (Table 13, 0.00625-day reference). Independently, one fixed graph seed per node-level scenario and policy is recomputed at Euler steps 0.1 and 0.05 day with unchanged 0.1-day controls. Maximum changes are 0.384% for peak and 0.396% for burden, smaller than the heterogeneity and churn effects.

## 4. Discussion

### 4.1. Why Coordination Works

Relationship suppression prevents exposure, whereas patching protects susceptible devices and removes infectious sources. Their effects enter multiplicatively in Equations (10) and (11), permitting capacity-limited actions to reinforce each other. JATP subsequently restores relationships as infection pressure declines; topology-specific Perron influence and independent trust evidence guide allocation.

The joint threshold crossing in Section 3.3 concerns the fully susceptible frozen-state index. The constant-control MFE threshold in Equation (10) additionally accounts for susceptible depletion by proactive patching.

### 4.2. Relation to Recent Control Research

Recent models have separately highlighted immediate response [15], awareness [28], distributed patch delay [18], mobile multilayer dynamics [34], and spatiotemporal feedback [33]. JATP could be extended to incorporate these mechanisms. Immediate response can be represented as a temporary increase in *u* or *v*; awareness modifies β or reporting quality; distributed patch delay can be added as a patch-availability state; mobile layers replace $A^{0}$ with a temporal operator; and spatial control can run below the stratum controller.

The joint formulation links logical relationship control, patch capacity, and service preservation. Within it, JATP offers inexpensive measurement-updated actions, whereas the offline benchmark quantifies the additional nominal performance attainable through horizon-wide optimization.

### 4.3. Deployment Architecture

The simulations use a centralized stratum controller with cached structural information and group-level commands. A hierarchical extension would assign local monitors to detection and flow collection, an estimator to ${\hat{E}}_{i}$ and ${\hat{I}}_{i}$, a central coordinator to Equation (27) and capacity enforcement, and local enforcers to patch and relationship actions. This architecture remains an implementation proposal.

This architecture aligns with self-adaptive trust management [11]. It also respects zero-trust principles: an established friendship is not perpetual authorization [44]. For privacy-sensitive domains, only stratum aggregates need to reach the controller. Local fog nodes can compute trust and infection estimates, while a central coordinator broadcasts budgets or multipliers.

### 4.4. Security–Service Interpretation

Retained weighted relationship intensity is not merely a regularization term. SIoT value depends on maintaining enough service-producing interactions while containment is active. Excessive suppression can increase discovery latency, concentrate load on remaining providers, and remove redundancy. The topology-only result illustrates the tension: it sacrifices substantially more weighted relationship intensity but still cannot repair existing infections. In contrast, additional patch capacity under JATP allows the relation layer to restore earlier.

Deployment-specific $w_{ij}^{\mathrm{svc}}$ and η should reflect service criticality. The graph experiment shows that connectivity can be maintained despite substantial loss of algebraic connectivity. Critical service pairs therefore need direct reachability or latency constraints in addition to the aggregate penalty in Equation (15).

### 4.5. Limitations and Threats to Validity

The three-stratum approximation is supported under the tested moderate heterogeneity and matched mixing. Strong degree variation and susceptible-entry churn require finer or open-population representations [8,34]. The explicit-network tests use deterministic mean-field probabilities on synthetic graphs; stochastic infection events and small-population extinction require event-level evaluation.

The experiments establish mechanism-level and comparative robustness under the specified uncertainty distribution, not estimates of absolute outbreak magnitude for Mirai, WannaCry, or another named family. Absolute peaks should therefore not be used for capacity planning without trace-based calibration. Trace-fitted self-propagation work provides a suitable parameter-inference template [7].

Trust scores are externally specified and fixed within each run. The synthetic trust-quality experiment characterizes how evidence alignment affects allocation; validation with observed SIoT trust and malware outcomes remains future work.

## 5. Conclusions

We introduced a malware-containment framework in which SIoT relationships and patch resources are controlled jointly. A controlled next-generation operator clarifies how protection, infectious duration, and logical topology interact. The optimal-control formulation makes service loss explicit, while the JATP feedback policy provides an implementable approximation based on stratum prevalence, topology-specific Perron influence, independent edge trust evidence, and service importance.

Under reference capacities, JATP improves containment relative to equally informed patch-only feedback, while the common-ceiling benchmark quantifies its objective excess over offline numerical optimization. The experiments identify how service costs, evidence quality, and network heterogeneity shape the gains and their limits. Trace-calibrated trust and finer representations of strongly heterogeneous or open populations remain priorities for deployment.

Logical relationships can serve as a reversible malware-control channel when capacities, service costs, and evidence quality are specified explicitly. The framework links topology and remediation analytically; its evaluation identifies useful operating conditions and limits requiring finer models, stronger estimation, or more intensive optimization.

## Figures and Tables

**Table 1:** Comparison of representative recent formulations. NR denotes a feature not established in the inspected source; NA denotes a feature outside the formulation.

Criterion	Liu et al. [45]	Cao et al. [34]	Guo et al. [33]	Su et al. [42]	Moeinaddini et al. [11]	This Study
Topology/granularity	Well-mixed intranet; host counts	Temporal layers; device/area	Spatial PDE; density	IoT nodes and communities	Dynamic SIoT; node/fog	Controlled weights; strata/node tests
Defense channels	Patch-test allocation	Patching; relation control NR	Patching and relation control NR	Provider selection; node exclusion	Malicious-node rejection; patching NA	Patching plus reversible weights
Trust information	No	NR	NR	Yes	Yes	External scores
Service criterion	Test and downtime costs	NR	NR	Service grades; provider selection	Service success	Retained intensity; graph checks
Feedback/solution	Open-loop PMP; iterative solution	Feedback NR; patch optimization	State feedback; designed law	Role-based trust updates	MAPE-K; ML/FL rules	Sampled feedback; heuristic plus separate NLP
Analytical threshold	NR	Invasion threshold	Turing/Hopf conditions	NA	NA	Reproduction threshold

PMP: Pontryagin maximum principle; PDE: partial differential equation; MAPE-K: monitor–analyze–plan–execute–knowledge; ML/FL: machine/federated learning; NLP: nonlinear programming. Liu et al. optimize the fraction of hosts assigned to patch testing, not the patching rate. Provider selection and node exclusion differ from continuous epidemic-edge suppression.

**Table 2:** Principal notation.

Symbol	Meaning
$N_{i}$	Normalized size of stratum *i*
$S_{i},E_{i},I_{i},R_{i}$	Susceptible, exposed, infectious, and recovered fractions
$a_{ij}^{0}$	Baseline relationship/contact intensity from *j* to *i*
$v_{ij}$	Reversible suppression applied to relationship class (i,j)
$u_{i}$	Patch/repair intensity in stratum *i*
$\beta_{i}$	Susceptibility-adjusted transmission coefficient
$\sigma_{i}$	Activation rate from exposed to infectious
$\gamma_{i}$	Native recovery rate
$\omega_{i}$	Immunity-loss rate
$B_{u},B_{v}$	Instantaneous patch and topology budgets
$Q_{\mathrm{svc}}\left(v\right)$	Retained weighted relationship intensity
$T_{ij},\theta_{ij}$	Edge trust evidence and trust deficit $1-T_{ij}$
η	Aggregate service-loss penalty weight
$\kappa_{T}$	Latent edge-risk amplitude in the trust experiment
$\chi_{T}$	Trust-quality coefficient in the trust experiment
$\psi_{i}^{S},\psi_{i}^{E},\psi_{i}^{I},\psi_{i}^{R}$	Adjoint variables
$R_{0},R_{\mathrm{inst}}$	Controlled MFE threshold and frozen-state reproduction index

**Table 3:** Numerical evaluation matrix.

Experiment	Levels/Replications	Purpose
Policy baseline	5 policies	Efficacy under channel-specific capacities
Common-ceiling/NLP	3 feedback policies; 2 starts and refinement	Shared expenditure bound and numerical optimization
Spectral grid	4 feasible patch × 4 feasible suppression levels	Joint threshold crossing
Patch budget	0.50–1.50 of baseline, 5 levels	Security–resource frontier
Contact stress	0.50–1.50, 11 levels	Outbreak-intensity robustness
Topology family	Balanced, community, hub, cross-coupled	Sensitivity across four synthetic matrices
Observation quality	13 delays × 3 noise levels × 10 seeds	Feedback robustness
Controller period	0.1–10 days, 9 levels	Sampled-data degradation and acceptable interval
Trust/service inputs	9 quality levels; 25 tuning points	Conditional information value and tuning sensitivity
Adversarial telemetry	Trusted and compromised feeds	Sensitivity to coordinated reporting bias
Parameter uncertainty	200 paired realizations	Distributional robustness
Explicit networks	3 sizes; 10 settings; 12 paired graphs	Renewal, churn, and strong heterogeneity
Graph service	Baseline; 3 controlled snapshots × 30 seeds	Connectivity, path stretch, and success
Controller scaling	K=3–128; $n=10^{3}$–$10^{6}$	Compute, communication, and aggregation cost
Local sensitivity	±10% for 5 parameter groups	Dominant mechanisms
Numerical convergence	5 RK4 steps	Discretization error

**Table 4:** Baseline comparison over 120 days. Infection variables are population fractions.

Policy	Peak *I*	Peak Day	∫Idt	Patch Effort	${\bar{Q}}_{\mathrm{svc}}$
No control	0.5158	25.5	19.5095	0	1.0000
Static patching	0.1344	26.8	4.7253	0.2430	1.0000
Risk-only patching	0.1273	28.0	4.7058	0.2490	1.0000
Topology-only	0.4458	30.9	18.5003	0	0.7333
JATP (proposed)	0.0644	29.7	2.8717	0.2503	0.8184

**Table 5:** Comparison under identical box limits, instantaneous capacities, and the common ceiling $C_{\mathrm{int}}\leq 0.1963840574$. Actual expenditure is reported, not assumed equal. All policies are evaluated with Equation (16) and the weights in Equation (32).

Policy	*J*	$E_{I}$	Peak *I*	I(T)	${\bar{Q}}_{\mathrm{svc}}$	$C_{\mathrm{int}}$
Risk-only	4.83896	4.70580	0.12734	0.00341	1.00000	0.092224
Topology-only	19.49265	18.55355	0.44817	0.05322	0.73593	0.196384
JATP	3.15064	2.87173	0.06439	0.00253	0.81841	0.196384
Direct NLP (10 days)	1.99341	1.69226	0.02503	0.00659	0.87958	0.196384
Direct NLP (5 days)	1.97461	1.67602	0.02482	0.00651	0.87919	0.196384

**Table 6:** Fully susceptible frozen-state reproduction index $R_{\mathrm{inst}}\left(N;u,v\right)$ over the budget-feasible constant-control grid at contact scale 0.18. Bold entries are below one.

		Relation Suppression			
		0	0.10	0.20	0.30
Patching	0.000	2.2445	2.0201	1.7956	1.5712
	0.015	1.8648	1.6783	1.4918	1.3053
	0.030	1.5982	1.4383	1.2785	1.1187
	0.045	1.3997	1.2597	1.1197	0.9798

**Table 7:** JATP security–resource frontier.

Patch Scale	Peak *I*	∫Idt	Patch Effort	${\bar{Q}}_{\mathrm{svc}}$	$B_{v}$ Active (%)
0.50	0.1900	8.2612	0.0633	0.7511	59.75
0.75	0.1114	5.0235	0.1414	0.7805	47.83
1.00	0.0644	2.8717	0.2503	0.8184	35.17
1.25	0.0396	1.6472	0.3896	0.8591	21.25
1.50	0.0270	1.0166	0.5593	0.8948	1.83

**Table 8:** Service-weight ablation. Both ${\bar{Q}}_{\mathrm{svc}}$ and $E_{v}^{\mathrm{ref}}$ are evaluated with the reference service weights, although the controller uses the stated input weights.

Variant	τ	Peak *I*	∫Idt	${\bar{Q}}_{\mathrm{svc}}$	$E_{v}^{\mathrm{ref}}$
Uniform weights	0.02800	0.06668	2.96273	0.81311	6.92684
Reference weights	0.02800	0.06439	2.87173	0.81841	7.12789

**Table 9:** JATP with 20% multiplicative observation noise (10 fixed-seed replications).

Delay (Days)	Mean Peak	Peak SD	Mean Burden	${\bar{Q}}_{\mathrm{svc}}$
0.0	0.06458	0.00016	2.87984	0.81889
0.5	0.06559	0.00018	2.90913	0.81889
1.0	0.06661	0.00020	2.93852	0.81891
2.0	0.06871	0.00023	2.99809	0.81897
4.0	0.07317	0.00025	3.12007	0.81924
6.0	0.07797	0.00025	3.24412	0.81961

**Table 10:** Paired Monte Carlo results over 200 parameter realizations.

Policy	Metric	Mean	SD	P05	P95
Risk-only	Peak	0.12849	0.02543	0.08408	0.16941
JATP	Peak	0.06639	0.01651	0.04041	0.09323
Risk-only	Burden	4.72181	0.70738	3.47769	5.83402
JATP	Burden	2.92552	0.62579	1.88910	3.94907
JATP	${\bar{Q}}_{\mathrm{svc}}$	0.81894	0.01252	0.79924	0.84145
JATP vs risk	Peak reduction (%)	48.829	2.778	44.236	52.675
JATP vs risk	Burden reduction (%)	38.642	4.367	31.781	45.644

**Table 11:** Paired inferential statistics over 200 common parameter draws. Differences are risk-only minus JATP, so positive values favor JATP.

Metric	Mean Diff.	Mean 95% CI	Median Diff.	Median Boot. CI	$d_{z}$	Wilcoxon *p*
Peak fraction	0.06210	[0.06081, 0.06340]	0.06271	[0.06158, 0.06425]	6.69	$7.18\;\times \;10^{-35}$
Burden	1.79629	[1.77801, 1.81457]	1.80863	[1.78239, 1.82698]	13.70	$7.18\;\times \;10^{-35}$

**Table 12:** Explicit-network JATP results with 12 graph realizations per setting. NRMSE compares the mean trajectory with the closed ODE, normalized by its peak. Churn and strong heterogeneity are reported as stress tests of the aggregate prediction.

*n*	Relationship Process	Mean Peak	Mean Burden	NRMSE (%)
100	Static	0.06824	2.96700	2.43
300	Static	0.06727	2.92944	1.83
1000	Static	0.06764	2.93991	2.06
100	Five-day renewal	0.06503	2.87607	0.61
300	Five-day renewal	0.06411	2.83953	0.68
1000	Five-day renewal	0.06450	2.85438	0.59
100	Renewal and 5% churn	0.06802	4.29839	22.74
300	Renewal and 5% churn	0.06587	4.19800	21.87
1000	Renewal and 5% churn	0.06625	4.23752	22.29
1000	High activity heterogeneity	0.10491	3.74677	25.44

**Table 13:** Numerical convergence of JATP relative to the 0.00625-day RK4 result with the control-update interval fixed at 0.1 day. Error columns are percentages.

RK4 Step	Peak	Burden	Peak Error (%)	Burden Error (%)
0.10000	0.06439416	2.87173091	$4.77\;\times \;10^{-4}$	$2.46\;\times \;10^{-6}$
0.05000	0.06439446	2.87173097	$7.65\;\times \;10^{-9}$	$5.94\;\times \;10^{-7}$
0.02500	0.06439446	2.87173098	$4.79\;\times \;10^{-10}$	$1.41\;\times \;10^{-7}$
0.01250	0.06439446	2.87173098	$2.90\;\times \;10^{-11}$	$2.81\;\times \;10^{-8}$
0.00625	0.06439446	2.87173098	0	0

## Data Availability

All data generated in this study, together with the simulation and plotting code, are provided in Supplementary File S1 and publicly mirrored in the GitHub repository (https://github.com/zhuxuejin/Malware-propagation-for-SIOT) (accessed on 22 September 2026). No proprietary or personal data were used.

## Supplementary Materials

The following supporting information can be downloaded at https://www.mdpi.com/article/10.3390/e28101078/s1, Supplementary File S1: a ZIP archive containing the Python 3.10 simulation and figure-generation code, dependency specification, fixed pseudorandom seeds defined in the simulator, 20 machine-readable CSV outputs, and seven generated vector PDF figures. The same materials are publicly available in the GitHub repository (https://github.com/zhuxuejin/Malware-propagation-for-SIOT) (accessed on 22 September 2026).

## Abbreviations

MFE	Malware-free equilibrium
IoT	Internet of Things
JATP	Joint adaptive topology and patching
MPC	Model predictive control
RK4	Fourth-order Runge–Kutta method
SEIR	Susceptible–exposed–infected–recovered
SIoT	Social Internet of Things

## Appendix A. Expanded Derivation of the Next-Generation Matrix

Order infected variables as $x={\left(E_{1},\ldots ,E_{K},I_{1},\ldots ,I_{K}\right)}^{T}$. At the malware-free equilibrium, the new-infection and transition Jacobians are

(A1) $$F=\left[\begin{array}{cc} 0 & F\\ 0 & 0 \end{array}\right],\;V=\left[\begin{array}{cc} \Sigma & 0\\ -\Sigma & D \end{array}\right].$$

The inverse of the block lower-triangular transition matrix is

(A2) $$V^{-1}=\left[\begin{array}{cc} \Sigma^{-1} & 0\\ D^{-1} & D^{-1} \end{array}\right].$$

Therefore

(A3) $$FV^{-1}=\left[\begin{array}{cc} FD^{-1} & FD^{-1}\\ 0 & 0 \end{array}\right].$$

Its nonzero eigenvalues are those of $FD^{-1}$, proving Equation (10). The activation matrix cancels because progression from $E_{i}$ to $I_{i}$ is certain. If exposed removal $q_{i}E_{i}$ is introduced, $\Sigma^{-1}$ is replaced by ${\left(\Sigma +Q\right)}^{-1}$ and the lower-left block becomes $D^{-1}\Sigma {\left(\Sigma +Q\right)}^{-1}$. The resulting threshold is

(A4) $$\rho \;\left(FD^{-1}\Sigma {\left(\Sigma +Q\right)}^{-1}\right),$$

which includes the probability of surviving exposed-stage detection.

## Appendix B. Adjoint Equations

For completeness, let

(A5) $$g_{i}=\beta_{i}\sum_{j}a_{ij}^{0}\left(1-v_{ij}\right)I_{j}/N_{j}.$$

The normal-cone terms affect control stationarity but not differentiation with respect to the state. The adjoint equations are therefore

(A6) $$\begin{array}{cc} {\dot{\psi }}_{i}^{S}= & -(\psi_{i}^{S}\left[-g_{i}-u_{i}\right]+\psi_{i}^{E}g_{i}+\psi_{i}^{R}u_{i}), \end{array}$$

(A7) $$\begin{array}{cc} {\dot{\psi }}_{i}^{E}= & -(-\sigma_{i}\psi_{i}^{E}+\sigma_{i}\psi_{i}^{I}),\\ {\dot{\psi }}_{i}^{I}= & -w_{i}+\left(\gamma_{i}+u_{i}\right)\psi_{i}^{I}-\left(\gamma_{i}+u_{i}\right)\psi_{i}^{R} \end{array}$$

(A8) $$\begin{array}{cc} \; & -\sum_{k}\left(\psi_{k}^{E}-\psi_{k}^{S}\right)\frac{\beta_{k}S_{k}a_{ki}^{0}\left(1-v_{ki}\right)}{N_{i}}, \end{array}$$

(A9) $$\begin{array}{cc} {\dot{\psi }}_{i}^{R}= & -\omega_{i}\psi_{i}^{S}+\omega_{i}\psi_{i}^{R}. \end{array}$$

These equations, the state system, terminal conditions, and the *C*- and $H_{v}$-metric conditions in Equations (23)–(25) define the necessary optimality system. A forward–backward sweep alternates state and adjoint integration to seek a stationary control candidate.

## Appendix C. Additional Parameter and Control Details

Table A1 collects the reference stratum parameters, initial conditions, and Perron influence values used by the simulations.

**Table A1:** Reference simulation values and interpretation.

Quantity	Endpoint	Gateway	Hub
Population $N_{i}$	0.50	0.32	0.18
Transmission $\beta_{i}$ (day^−1^)	0.44	0.52	0.61
Activation $\sigma_{i}$ (day^−1^)	0.28	0.24	0.20
Native recovery $\gamma_{i}$ (day^−1^)	0.055	0.070	0.090
Immunity loss $\omega_{i}$ (day^−1^)	0.006	0.006	0.005
Perron influence (mean one)	0.6967	1.0644	1.2389
Initial exposed	0.0010	0.0007	0.0004
Initial infectious	0.0020	0.0012	0.0008

Risk-only and JATP use the population-weighted capacity $\sum_{i}N_{i}u_{i}\leq 0.045$ day^−1^ with per-stratum cap ${\bar{u}}_{i}=0.15$ day^−1^ and capped redistribution. Static patching applies $u_{i}=0.045$ day^−1^, exactly exhausting the same population-weighted budget. The raw relation response is

(A10) $${\tilde{v}}_{ij}=0.72\frac{p_{ij}}{p_{ij}+0.028\delta_{ij}},$$

followed by the projection in Equation (27). The cap 0.72 retains at least 28% of each relationship class; τ=0.028 sets the reference saturation scale. Exposed-state discounts $\xi_{u}=0.35$ and $\xi_{v}=0.25$ reduce latent-source contributions, while $\alpha_{T}=1$ permits trust to double pair risk. These illustrative settings, $T^{0}$, and $W^{\mathrm{svc}}$ are fixed except in the designated sensitivity experiments. Perron influence and $h_{ij}$ are recomputed for each topology or perturbed matrix. The trust experiment uses Equations (33)–(35), with reference $\kappa_{T}=2$ permitting an unnormalized risk ratio up to three and an amplitude sweep over {0.5,1.0,1.5,2.0}. The tuning grid varies τ and $\alpha_{T}$ while retaining the exposed-state discounts.

## Appendix D. Complete Observation-Robustness Grid

Table A2 gives the complete observation-delay and noise grid underlying the selected values in Table 9 and Figure 9.

Delay dominates independent noise throughout the tested zero-to-six-day range. This follows from the epidemic growth phase: a delayed controller systematically reacts to an earlier, smaller state, whereas zero-mean multiplicative errors partially average across strata and epochs. The explicit adversarial model in Section 3 confirms the greater impact of coordinated hub under-reporting.

**Table A2:** Mean peak infection for all observation-delay and noise combinations.

Delay	No Noise	10% Noise	20% Noise
0.0	0.06439	0.06445	0.06458
0.5	0.06540	0.06545	0.06559
1.0	0.06642	0.06647	0.06661
1.5	0.06746	0.06751	0.06765
2.0	0.06852	0.06857	0.06871
2.5	0.06961	0.06966	0.06979
3.0	0.07071	0.07077	0.07090
3.5	0.07184	0.07190	0.07203
4.0	0.07299	0.07305	0.07317
4.5	0.07416	0.07421	0.07434
5.0	0.07535	0.07541	0.07554
5.5	0.07656	0.07662	0.07675
6.0	0.07779	0.07784	0.07797
